# Different Roles for the Axin Interactions with the SAMP versus the Second Twenty Amino Acid Repeat of Adenomatous Polyposis Coli

**DOI:** 10.1371/journal.pone.0094413

**Published:** 2014-04-10

**Authors:** Jean Schneikert, Jan Gustav Ruppert, Jürgen Behrens, Eva Maria Wenzel

**Affiliations:** 1 Nikolaus-Fiebiger-Center for Molecular Medicine, University of Erlangen-Nürnberg, Erlangen, Germany; 2 Centre for Cancer Biomedicine, Faculty of Medicine, University of Oslo, Oslo, Norway; Northwestern University Feinberg School of Medicine, United States of America

## Abstract

Wnt signalling is prevented by the proteosomal degradation of β-catenin, which occurs in a destruction complex containing adenomatous polyposis coli (APC), APC-like (APCL), Axin and Axin2. Truncating mutations of the APC gene result in the constitutive stabilisation of β-catenin and the initiation of colon cancer, although tumour cells tolerate the expression of wild-type APCL. Using the colocalisation of overexpressed Axin, APC and APCL constructs as a readout of interaction, we found that Axin interacted with the second twenty amino acid repeat (20R2) of APC and APCL. This interaction involved a domain adjacent to the C-terminal DIX domain of Axin. We identified serine residues within the 20R2 of APCL that were involved in Axin colocalisation, the phosphorylation of truncated APCL and the down-regulation of β-catenin. Our results indicated that Axin, but not Axin2, displaced APC, but not APCL, from the cytoskeleton and stimulated its incorporation into bright cytoplasmic dots that others have recognised as β-catenin destruction complexes. The SAMP repeats in APC interact with the N-terminal RGS domain of Axin. Our data showed that a short domain containing the first SAMP repeat in truncated APC was required to stimulate Axin oligomerisation. This was independent of Axin colocalisation with 20R2. Our data also suggested that the RGS domain exerted an internal inhibitory constraint on Axin oligomerisation. Considering our data and those from others, we discuss a working model whereby β-catenin phosphorylation involves Axin and the 20R2 of APC or APCL and further processing of phospho-β-catenin occurs upon the oligomerisation of Axin that is induced by binding the SAMP repeats in APC.

## Introduction

Homeostasis of the colonic epithelium requires the proliferation of the stem cells located at the bottom of the crypts, the subsequent expansion of the daughter cell population, the differentiation and migration of these cells toward the surface and ultimately apoptosis and release into the lumen [1]. These processes are partly coordinated by Wnt family growth factors that encourage cell proliferation. This increase in cell proliferation occurs upon the accumulation of the transcription factor β-catenin [2], [3], which controls a genetic program at the origin of cell proliferation. In the absence of Wnt stimulation, β-catenin is targeted for degradation in a destruction complex consisting of the tumour suppressor APC bound to Axin or Axin2, which interacts with casein kinase 1α (CK1α) and glycogen synthase kinase 3β (GSK3β) [4], [5]. The phosphorylation of β-catenin catalysed by these kinases generates a signal for the subsequent ubiquitination of phosphorylated β-catenin, followed by proteasomal degradation. Several models have been proposed to concatenate the currently available data [6]–[9]. The most recent model opposes the classical view of Wnt-elicited disruption of the destruction complex based on evidence that the destruction complex remains essentially intact upon Wnt stimulation [9]–[11].

**Figure 1:**
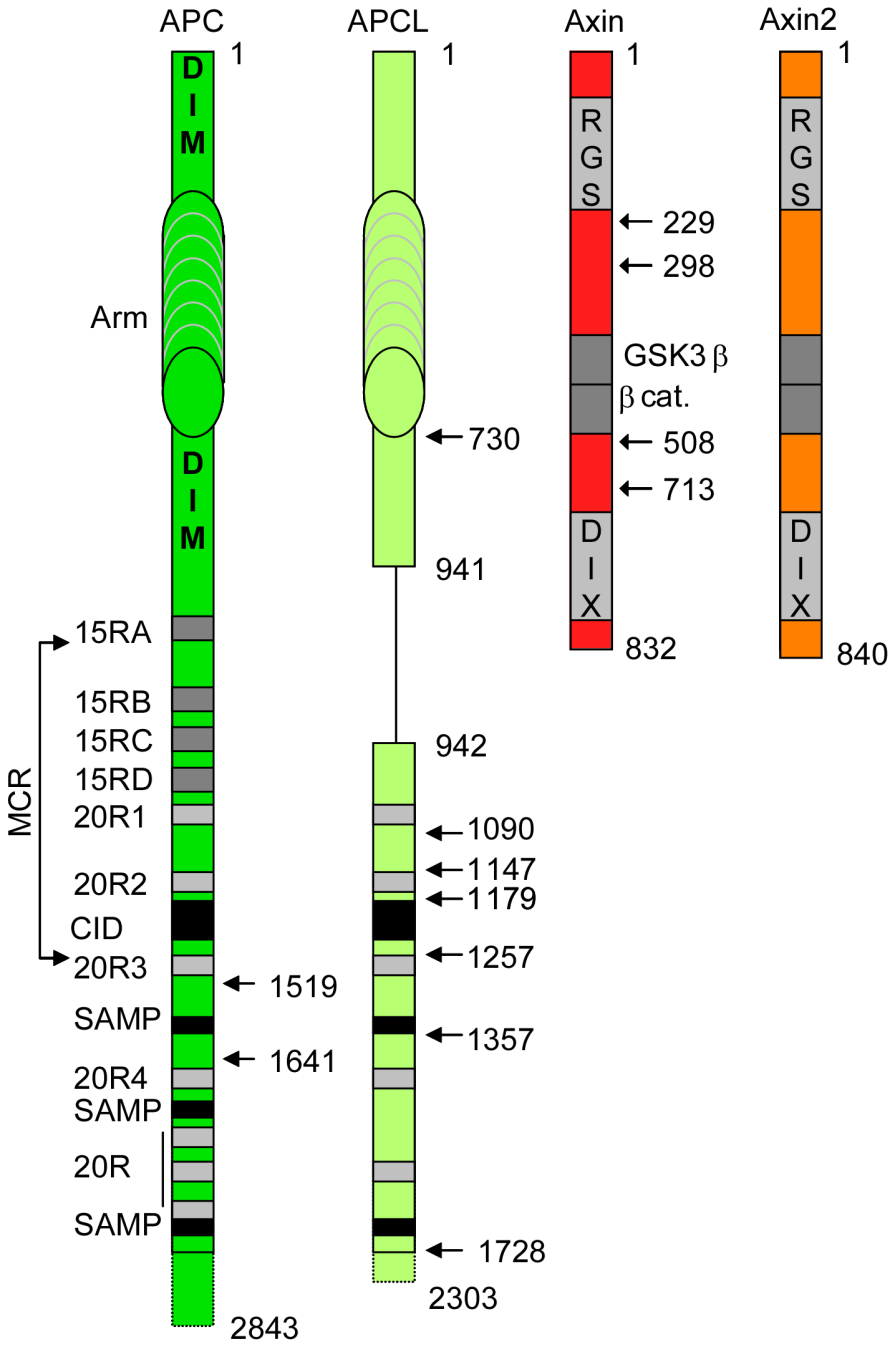
Schematic representation of human APC and APCL and rat Axin. Functional modules are shown, including the two dimerisation domains of APC (DIM), the armadillo repeat domain (Arm), the 15 (15RA to D) and 20 (20R1 to 7) amino acid repeats that function as β-catenin binding sites, the β-catenin inhibitory domain (CiD) involved in β-catenin degradation and the SAMP repeats that represent Axin or Axin2 binding sites. The mutation cluster region (MCR) contains most of the APC truncating mutations that have been observed in colon cancer. The RGS (SAMP-binding region), DIX (oligomerisation), β-catenin and GSK3β domains in Axin are indicated. The numbers indicate the amino acid positions. The numbers with arrows correspond to the size of the different constructs analysed in this study, which were fused at the N-terminus to YFP (APC and APCL) or myc (Axin).

The mechanistic steps that involve APC are not completely understood. APC is a large protein of 2843 amino acids (**fig. 1**) [12], [13]. The long N-terminal region contains two dimerisation domains [14], [15] that bracket the armadillo repeat domain, which is followed by β-catenin-binding sites termed the 15 [16]–[18] and 20 [19] amino acid repeats (15R and 20R, respectively); the 20Rs are intermingled with the β-catenin inhibitory domain (CiD) [8], [20] and the SAMP repeats that are Axin/Axin2-binding sites [21], [22]. The C-terminus of APC is involved in microtubule dynamics, and it is not known whether this region plays a role in β-catenin degradation [23]. The 15R bind to β-catenin and are important for targeting β-catenin for degradation [24]. The 20R are heterogeneous; the 20R1 and 20R3 bind to β-catenin with differing affinities [18], but only the 20R3 has been implicated in β-catenin degradation [24], [25]. The role of the 20R1 remains unknown [24]. Paradoxically, the 20R2 cannot bind to β-catenin [18]. The CiD likely constitutes an interaction surface for a component crucial for β-catenin degradation [8], [20].

APC has received particular attention because truncating mutations have been identified in approximately 80% of colorectal tumours [26]. The mutations tend to cluster in the middle of the open reading frame; this region has therefore been termed the mutation cluster region (MCR) [27]. This clustering reflects simultaneous positive and negative selection processes [28]. On one hand, the mutations remove the SAMP repeats, a nuclear export signal [29], the 20R3 [30] and the CiD [20], all domains known to contribute to β-catenin degradation. As a consequence, these mutations stabilise β-catenin and shift the homeostatic balance toward cell proliferation and the initiation of tumourigenesis. On the other hand, truncating mutations almost always occur after the first 15R [24], [31]. The resulting fragment may have multiple roles because the armadillo repeat domain binds to several partners [32]–[36]. The strong selection for the presence of the first 15R in truncated APC suggests that it serves an important purpose.

Besides APC, colorectal cancer cells also express the paralogue APC-like (APCL) [37]–[39]. APC and APCL display similar topological organisation and share most of the functional domains, with the exception of the 15R region, the second dimerisation domain and the C-terminus (**fig. 1**). APCL targets β-catenin for destruction upon ectopic expression [37]–[38], and it is puzzling that sequence alterations in APCL have yet to be reported in colon cancer.

Axin has been proposed to be the limiting component of the β-catenin destruction complex because it is present at extremely low concentrations in Xenopus extracts [40]. The evidence that APC and Axin or Axin2 interact via the SAMP repeats stems from studies that analysed the contribution of the SAMP repeats to β-catenin degradation using APC constructs lacking approximately the N-terminal thousand amino acids including the 15R [25], or containing point mutations in the 15R that abolish β-catenin binding [24]. Based on these results, it was thought that Axin and Axin2 catalyse β-catenin degradation by binding to the SAMP repeats of APC. However, this is only partly satisfying because the ectopic expression of Axin or Axin2 in colorectal cancer cell lines expressing APC that is truncated before the SAMP repeats results in efficient β-catenin destruction [21], [41], [42]. Furthermore, ectopic expression of truncated APC lacking all the SAMP repeats also elicits efficient β-catenin degradation [20]. Both results create a paradox as they do not reveal any dependent relationship between Axin and its ability to bind to the SAMP repeats of APC to elicit β-catenin degradation.

Axin oligomerises through its DIX domain (**fig. 1**); this oligomerisation is observed as bright cytoplasmic spots upon ectopic Axin expression [43]–[46]. Interestingly, ectopically expressed Axin lacking the DIX domain exhibits a diffuse intracellular distribution and results in the accumulation of phosphorylated β-catenin that does not undergo subsequent degradation [43]. This indicates that Axin oligomers represent the fully active destruction complex. It has also been shown that APC is essential for destruction complex assembly, that APC provokes Axin oligomerisation and that Wnt stimulation leads to Dishevelled activation, which oligomerises with Axin and prevents it from oligomerising with APC [45], [46]. In addition, it has been demonstrated that the destruction complex catalyses the ubiquitination of phosphorylated β-catenin; this ubiquitination event is inhibited by Wnt stimulation [9]. Together, these data suggest that Axin binding to APC results in Axin oligomerisation that is associated with the ubiquitination of phosphorylated β-catenin; all of these steps are inhibited by Wnt stimulation as a result of Dishevelled activation. However, this model does not convey how β-catenin phosphorylation is related to the APC/Axin interaction despite observations that both proteins individually stimulate β-catenin phosphorylation [43], [47]. In addition, this model does not provide an answer to the contradictory observations that the Axin/APC interaction is either not perturbed [9] or is affected [48], [49] by Wnt stimulation.

The study presented here aimed to solve this paradox by providing evidence that Axin performs a different function when bound to the second twenty amino acid repeat (20R2) of APC than when bound to the SAMP repeats of APC.

## Results

### APC and APCL differentially influence the intracellular localisation of Axin

We analysed the interaction of Axin with both APC and APCL in colocalisation experiments using N-terminal tagged constructs (**fig. 1**) that were transiently transfected into colorectal cancer cell lines. We used three different Axin expression constructs, mouse flag-Axin (fAxin), rat myc-Axin (mycAxin) and human YFP-Axin (yAxin). When the three constructs were transiently expressed in DLD1 or SW480 cells, we observed two intracellular localisation patterns for all three constructs, a diffuse distribution and a dotty cytoplasmic distribution (**fig. S1A**); the proportion of these patterns varied between constructs and cell lines (**fig. S1B**). The dotty pattern is commonly observed upon ectopic expression of Axin [50], [51] and has also been described for endogenous Axin [52], [53]. The dots are oligomers [44] that form spontaneously as a function of increasing Axin concentration [45]. The different proportions of the two localisation patterns in a given cell line may therefore reflect differences in the expression levels of the three constructs based on the species of origin of the construct and/or the influence of the tag on Axin oligomerisation. For a given construct, the different proportion of Axin localisation between two cell lines may reflect different expression levels resulting from different transfection efficiencies. We established that the transfection efficiency is better in SW480 cells than in DLD1 cells [unpublished data]. Meanwhile, the careful inspection of any cell line transfected with any construct revealed that many cells expressed higher levels of diffuse Axin and other cells exclusively displayed discrete dots. This indicates that additional factors influence Axin oligomerisation.

**Figure 2:**
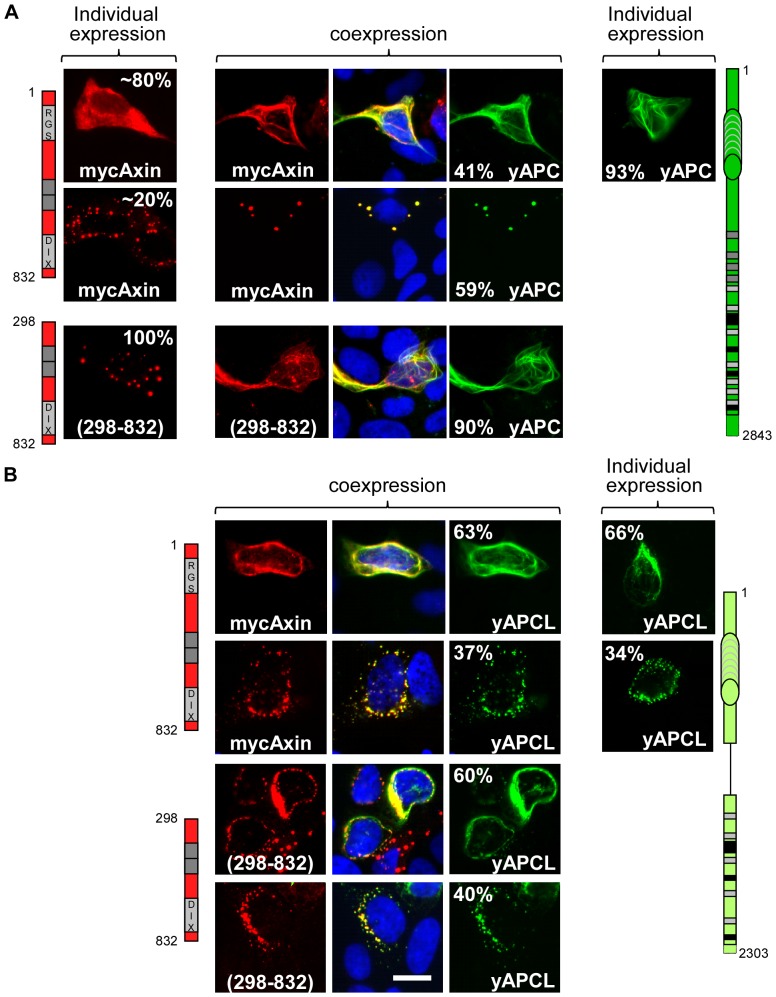
The RGS domain of Axin is not necessary for colocalisation with APC or APCL. DLD1 cells were transiently transfected on day 1 with the indicated APC (**A**) or APCL (**B**) constructs or with the indicated N-terminal myc-tagged Axin constructs, either individually or in combination. The cells were stained on day 3 with an anti-myc antibody and Hoechst dye. Cells transfected with yAPCL alone displayed dotty and fibre-like expression patterns, with yAPC alone exhibited a fibre-like expression pattern, with myc-Axin alone displayed diffuse and dotty expression patterns and with myc-Axin(298–832) alone exhibited only a dotty expression pattern. Coexpression of myc-Axin and APC or APCL resulted in colocalisation, which was also observed with when the RGS domain (298–832) was deleted from Axin. The percentages indicate the proportion of different localisation patterns in the transfected cells. The imaging parameters were identical for the myc constructs; the yAPCL signal was more intense than the yAPC signal. Bar, 10 μM.

When individually expressed in DLD1 cells, YFP-labelled APC (yAPC) predominantly exhibited a fibre-like pattern (**fig. 2A**), likely corresponding to colocalisation with microtubules, as has been previously described [54], [55]. We confirmed the fortuitous observation that myc-Axin was diffusely localised in the cytoplasm of most transfected DLD1 cells (approximately 80%). Co-expression of myc-Axin and yAPC resulted in two predominant observations, either the recruitment of myc-Axin to fibre-bound yAPC (41% of myc-Axin- and yAPC-positive cells) or the re-localisation of yAPC and myc-Axin to bright spots (59% of myc-Axin- and yAPC-positive cells). Thus, yAPC and myc-Axin mutually stimulate the recruitment of one another into dots that represent the β-catenin destruction complex, or the degradasome [43]–[46]. When individually expressed in DLD1 cells, YFP-labelled APCL (yAPCL) displayed a fibre-like pattern in approximately two-thirds of the transfected cells (**fig. 2B**), likely corresponding to colocalisation with microtubules and/or actin fibres, as has been previously described [39]. In the remaining one-third of the transfected cells, yAPCL localised to bright punctuate structures surrounding the nucleus. Cells exhibiting both types of localisation were rare (approximately 10% of the transfected cells) and were counted as the dotty type. In contrast to yAPC, the addition of myc-Axin neither increased the proportion of cells containing yAPCL dots nor decreased the fraction displaying fibre-bound yAPCL. Axin was no longer diffusely localised and instead colocalised with both types of yAPCL, which may also have been interacting with endogenous Axin. Notably, there was no correlation between the different intracellular localisation patterns and the expression level of each construct: we commonly identified numerous cells with high levels of diffuse myc-Axin next to cells with few and discrete myc-Axin dots as well as the inverse. These observations were also common to yAPCL and the other constructs in all the subsequent experiments. We concluded that myc-Axin and yAPC mutually stimulated the incorporation of one another into dots, whereas yAPCL imposed its own intracellular localisation pattern on myc-Axin.

### The N-terminus of Axin, which contains the RGS domain, may inhibit oligomerisation

The well-known interaction between Axin and APC occurs via the N-terminal RGS domain and the SAMP repeats (**fig. 1**). Deletion of the RGS domain (myc-Axin(298–832); **fig. 2**) resulted in a transition from a diffuse localisation pattern to a fully penetrant dotty cytoplasmic pattern. This probably reflected Axin oligomerisation because these dots disappeared upon deletion of the DIX domain that mediates oligomerisation (see below). We concluded that the N-terminus of Axin, which contains the RGS domain, may have inhibited Axin oligomerisation.

### The colocalisation of Axin with APC or APCL is independent of the RGS

Surprisingly, the co-expression of yAPC and myc-Axin(298–832) (**fig. 2**) resulted in the partial elimination of myc-Axin(298–832) dots and in the recruitment of myc-Axin(298–832) to fibre-bound yAPC. Similarly, myc-Axin(298–832) colocalised with yAPCL, despite lacking the RGS domain. We concluded that Axin may contain a second interaction domain for both APC and APCL that is independent of the RGS-SAMP interface.

### yAPCL1728 dots are not Golgi vesicles

**Figure 3:**
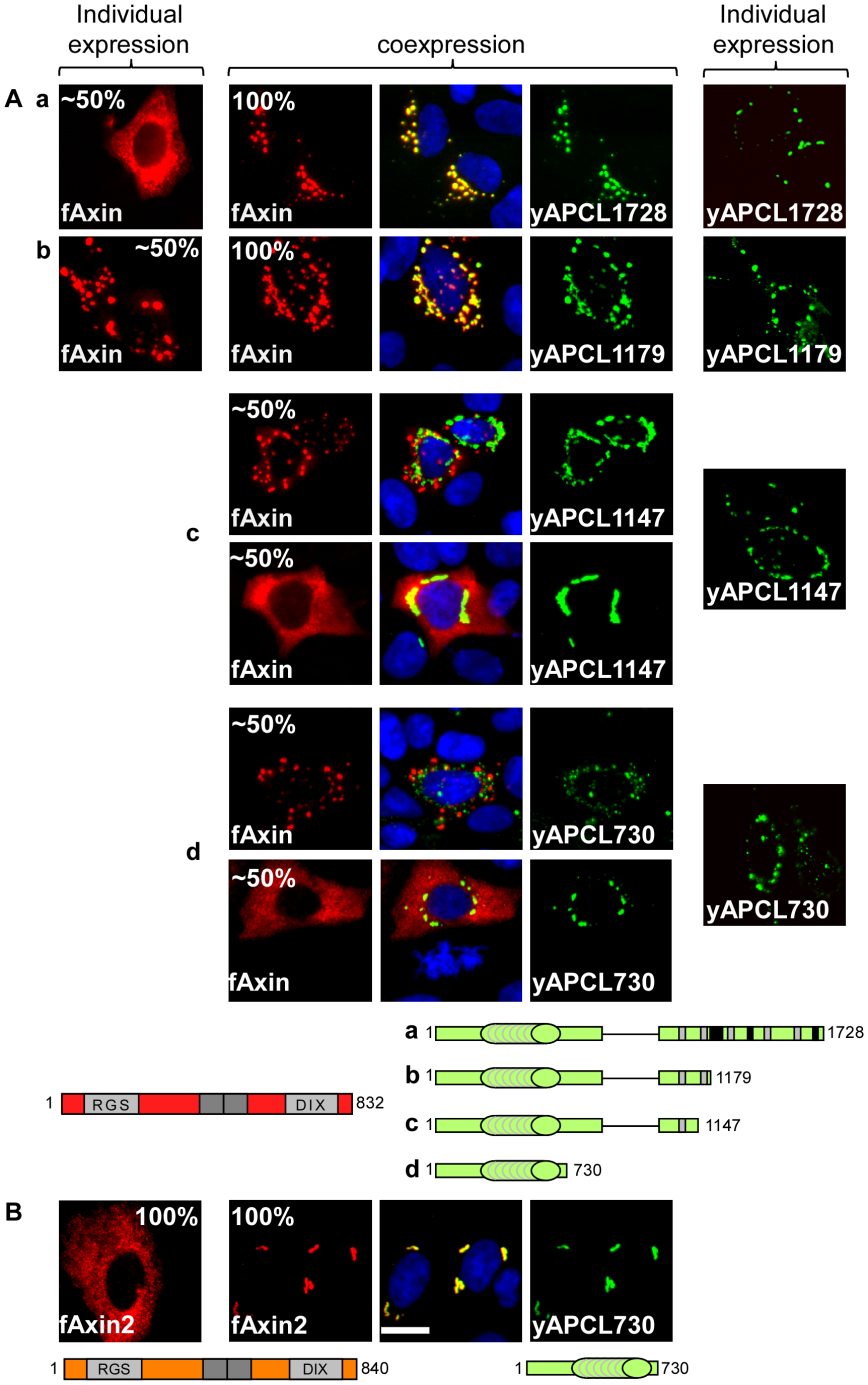
The 20R2 of APCL is required for Axin colocalisation in the absence of the SAMP repeats. DLD1 cells were transiently transfected on day 1 with the indicated APCL constructs or the indicated N-terminal flag-tagged Axin (**A**) or Axin2 (**B**) constructs, either individually or in combination. The cells were stained on day 3 with an anti-flag antibody and Hoechst dye. When expressed alone, truncated yAPCL displayed a dotty localisation pattern, flag-Axin exhibited a diffuse or dotty expression pattern and fAxin2 demonstrated diffuse localisation. **A**, APCL constructs lacking the 20R2 (i.e., 1147 and shorter) did not colocalise with fAxin (**c**, **d**), whereas colocalisation occurred in the presence of the 20R2 (yAPCL1179 and yAPCL1728) (**a**, **b**). **B**, Axin2 interacted with the N-terminus of APC. The percentages indicate the proportion of different localisation patterns in the transfected cells. The imaging parameters were identical for each type of tag. Bar, 10 μM.

In a first step toward identifying the domains involved in this putative novel Axin-APC(L) interaction, we utilised yAPCL constructs progressively deleted from the C-terminus (**fig. 1**). These constructs localised exclusively as cytoplasmic dots when transiently expressed in the DLD1 colorectal cancer cell line (**fig. 3**). Fibre localisation was lost upon deleting the C-terminus, which likely contains a microtubule-binding site based on analogy to the C-terminus of APC [23], [39]. The punctuate structures were typically restricted to the vicinity of the nucleus and may have represented Golgi vesicles, as has been previously described [39]. To investigate this possibility, SW480 cells transiently expressing yAPC1728 were probed with antibodies against the Golgi markers GM130 [56], giantin [57] and TGN46 [58] (**fig. S2**). Although displaying a very similar appearance as yAPCL1728, none of the Golgi markers colocalised with yAPCL1728. Therefore, we concluded that the yAPCL1728 dots were not Golgi vesicles.

### The 20R2 of truncated APCL is necessary for Axin colocalisation

In contrast to myc-Axin, N-terminal flag-tagged Axin (flag-Axin) was expressed as cytoplasmic dots in about half of the transfected cells (**fig. 3A** **, S1**). Co-expression of yAPCL1728 and flag-Axin resulted in colocalisation of the two proteins (**fig. 3A**). Deletion of both SAMP repeats (yAPCL1179 or yAPCL1257 [unpublished data]) did not affect APCL/Axin colocalisation, suggesting the presence of an Axin-binding site in APCL that does not require the SAMP repeats. Importantly, deletion of the second 20 amino acid repeat (20R2) eliminated the colocalisation (compare yAPCL1179 with yAPCL1147, **fig. 3A**). When individually expressed, flag-Axin displayed a diffuse cytoplasmic localisation in approximately half of the cell population (**fig. 3A** **, S1**). The diffuse flag-Axin pattern was retained only upon co-expression of APCL constructs lacking the 20R2. Similar results were obtained using myc-Axin instead of flag-Axin [unpublished data]. We concluded that Axin colocalisation with truncated APCL required the 20R2.

### Axin2 colocalises with the N-terminus of APCL

We performed similar experiments with N-terminal flag-tagged Axin2 (fAxin2), which exhibited a diffuse cytoplasmic distribution in DLD1 cells upon ectopic expression (**fig. 3B**). Surprisingly, APCL constructs as short as yAPCL730 (**fig. 1**) colocalised with flag-Axin2. Therefore, flag-Axin2 colocalised specifically with the N-terminus of APCL, whereas Axin colocalisation with APCL was lost when the 20R2 was deleted.

### Amino acids 508-712 of Axin are required for colocalisation with APCL truncated before the SAMP repeats

**Figure 4:**
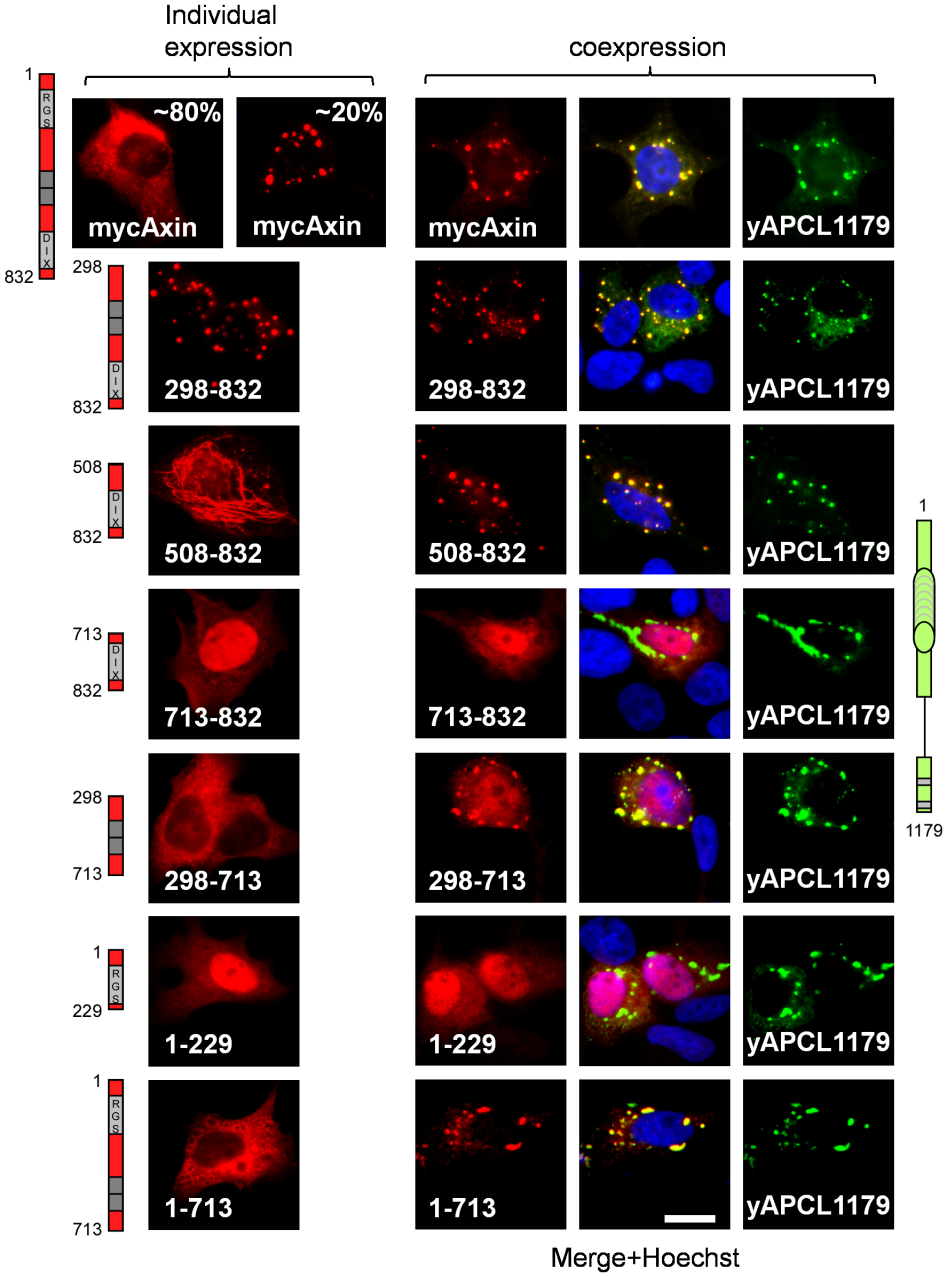
Amino acids 508–713 of Axin are required for colocalisation with truncated APCL. DLD1 cells were transiently transfected on day 1 with wild-type or mutant N-terminal myc-tagged Axin, either individually or in combination with the yAPCL1179 expression vector. The cells were fixed on day 3 and were stained with an anti-myc antibody and Hoechst dye. The presence of amino acids 508–713 in the myc-Axin deletion mutants resulted in colocalisation with yAPCL1179. Where applicable, the percentages indicate the proportion of different localisation patterns in the transfected cells. The imaging parameters were identical for each type of tag. Bar, 10 μM.

We also performed the converse experiment, using yAPCL1179 truncated shortly after the 20R2 and deletion mutants of myc-Axin, to identify a putative second APCL-binding site in Axin that was independent of the RGS domain (**fig. 4**). We first confirmed that myc-Axin and myc-Axin(298–832), which lacks the RGS domain, colocalised with yAPCL1179 (**fig. 4**). Surprisingly, further N-terminal deletions in myc-Axin (myc-Axin(508–832)) resulted in a fibre-like localisation pattern of Axin when individually expressed, but the colocalisation with yAPCL1179 in dots remained. The colocalisation was lost upon deletion of residues 508 to 712 (myc-Axin(713–832)). When individually expressed, myc-Axin(713–832) was diffusely distributed throughout the cell. The RGS domain alone (myc-Axin(1–229)) did not colocalise with yAPCL1179, whereas deletions of either the DIX domain (myc-Axin(1–713)) or the DIX and RGS domains (myc-Axin(298–713)) did not affect Axin colocalisation with yAPCL1179 (**fig. 4**). We concluded that Axin colocalisation with yAPCL1179 depended on amino acids 508 to 712 of Axin, thereby suggesting the presence of an APCL-binding site that was independent of the RGS domain.

### Conserved residues within the 20R2 are required for truncated APCL to colocalise with Axin

**Figure 5:**
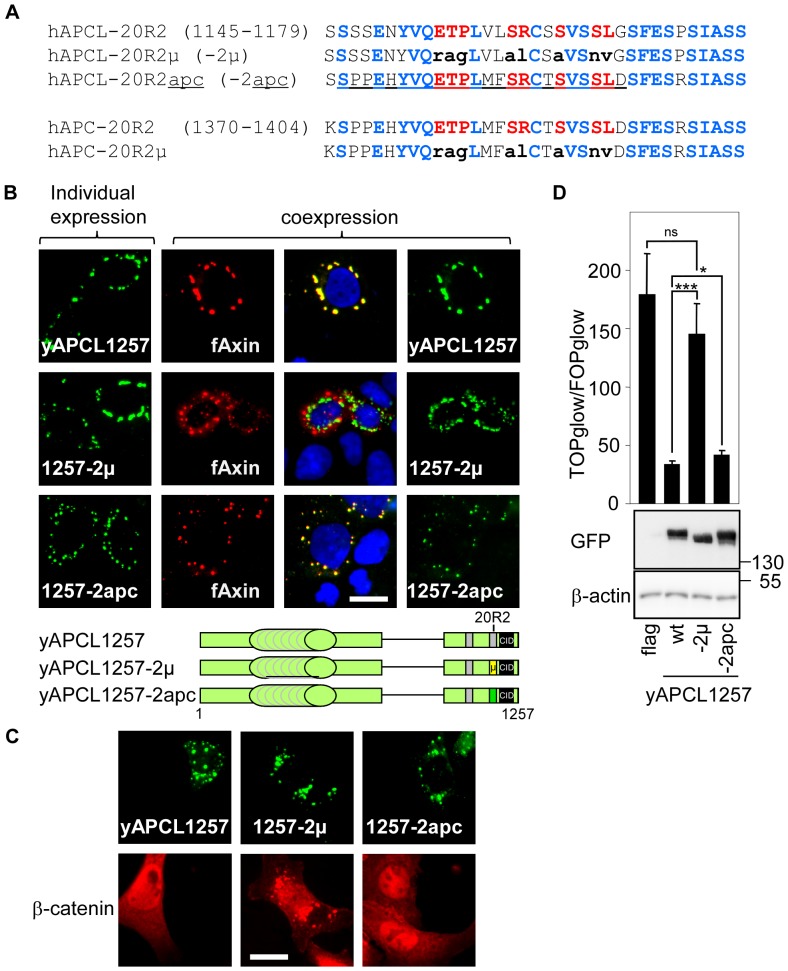
The 20R2s of APC and APCL mediate Axin colocalisation, β-catenin down-regulation and the inhibition of β-catenin transcriptional activity in the absence of the SAMP repeats. A, 20R2 sequences of wild-type and mutant human APC (residues 1370–1404) and APCL (residues 1145–1179). Red indicates amino acids common to APC and APCL from various species (fig. S2), blue represents the residues common to human APC and APCL, and the small bold black letters signify the residues that have been mutated. The underlined sequence comes from the human APC 20R2. B, Mutation of the 20R2 abolished the colocalisation of APCL with Axin, whereas the 20R2 of APC restored colocalisation with Axin. DLD1 cells were transiently transfected on day 1 with the indicated APCL constructs or N-terminal flag-tagged Axin, either individually or in combination. The cells were fixed on day 3 and were stained with an anti-flag antibody and Hoechst dye. Bar, 10 μM. C, SW480 cells were transiently transfected with a control vector (flag) or the indicated APCL constructs, fixed on day 3 and stained with an anti-β-catenin antibody. Bar, 10 μM. D, SW480 cells were transiently transfected on day 1 with reporter plasmids and 100 ng of an empty vector (flag) or the indicated APCL constructs. TOP/FOP reporter assays were performed on day 3 to measure β-catenin transcriptional activity. The data are presented as the mean ± standard deviation of three independent values from a representative experiment. * p<0.05, *** p<0.001, ns, not significant using Student's t-test. In a parallel experiment, cells were transiently transfected with 1 μg of the indicated plasmids on day 1. Cell extracts were prepared on day 3 and subjected to western blotting using the indicated antibodies. The molecular weights are shown in kDa. The imaging parameters were identical for each type of tag.

To provide additional evidence that Axin binds to the 20R2 of APCL, we compared the 20R2 sequences of APC and APCL from different species (**fig. S3**). We identified eight amino acids common to all inspected 20R2 sequences. These eight residues were mutated in the context of yAPCL1257 (yAPCL1257-2μ, **fig. 5A**). Co-expression of yAPCL1257-2μ and flag-Axin in DLD1 cells revealed that the mutations abolished the colocalisation of these two proteins (**fig. 5B**). We concluded that the conserved residues within the 20R2 were required for truncated APCL to colocalise with Axin. Next, we removed the 1090 N-terminal residues from yAPCL1257 and yAPCL1257-2μ to create two constructs, yAPCL(1091–1257) and yAPCL(1091–1257)-2μ, which exhibited a diffuse cytoplasmic distribution (**fig. S4**). The expression of yAPCL(1091–1257) dissolved the flag-Axin dots, whereas the expression of yAPCL(1091–1257)-2μ did not affect these dots. Together, we concluded that the conserved residues within the 20R2 were required for truncated APCL to colocalise with Axin, that the N-terminal residues of APCL were not required and that colocalisation with the 20R2 inhibited Axin oligomerisation.

### The 20R2 of APC can replace that of APCL to enable truncated APCL to colocalise with Axin

To determine whether the 20R2 of APC also colocalised with Axin, we exchanged the 20R2 of APCL with the corresponding sequence of its paralogue (yAPCL1257-2apc, **fig. 5A**). The 20R2 of APC could replace that of APCL to enable colocalisation with flag-Axin (**fig. 5B**).

### The 20R2 of truncated APCL is required for β-catenin down-regulation and can be replaced with the 20R2 of APC

We analysed the functional consequences of mutating the 20R2 of APCL or replacing it with that of APC. Immunofluorescence experiments were performed in SW480 cells, in which β-catenin is easily detectable, enabling its down-regulation upon the expression of either APC or APCL to be observed. Mutating the 20R2 of APCL in the context of the active yAPCL1257 (yAPCL1257-2μ) abolished β-catenin down-regulation and led to the colocalisation of APCL with β-catenin (**fig. 5C**). The 20R2 of APC (yAPCL1257-2apc) could substitute for that of APCL (**fig. 5C**). yAPCL1257 and yAPCL1257-2apc, but not yAPCL1257-2μ, inhibited the TOP reporter that measures β-catenin transcriptional activity (**fig. 5D**). We concluded that β-catenin degradation by truncated APCL correlated with Axin colocalisation to the 20R2 in the absence of the SAMP repeats.

### Identifying the residues in the 20R2 that are involved in phosphorylating truncated APCL

**Figure 6:**
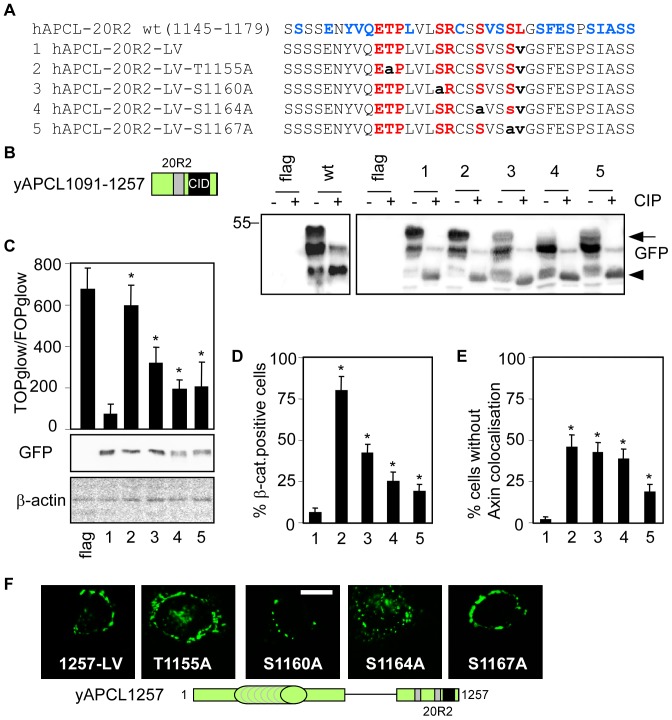
The residues of the 20R2 required for APCL phosphorylation are also required to inhibit β-catenin transcriptional activity, to target β-catenin for degradation and to interact with Axin. A, The sequences of the wild-type and mutant 20R2 from APCL (residues 1145–1179). The constructs are numbered in B–E. Bold capital letters indicate residues common to APC and APCL from various species (fig. S2), and small bold letters signify residues that have been mutated. B, Amino acids within the 20R2 that were involved in the phosphorylation of truncated APCL. 293T cells were transiently transfected on day 1 with the indicated yAPCL(1091–1257) constructs. On day 3, cell extracts were subjected to immunoprecipitation using an anti-GFP antibody followed by alkaline phosphatase treatment (CiP) and western blotting using the anti-GFP antibody. The arrow indicates the phosphorylated APCL fragments that were affected by mutation, and the arrowhead points to non-phosphorylated APCL constructs. C, SW480 cells were transiently transfected on day 1 with reporter plasmids and 100 ng of an empty vector (flag) or the indicated yAPCL1257 constructs. TOP/FOP reporter assays were performed on day 3 to measure β-catenin transcriptional activity. The data are presented as the mean ± standard deviation of three independent values from a representative experiment. * p<0.05 compared with mutant number 1, Student's t-test. In a parallel experiment, cells were transiently transfected with 1 μg of the indicated plasmids on day 1. Cell extracts were prepared on day 3 and were subjected to western blotting using the indicated antibodies. The molecular weights are shown in kDa. D, SW480 cells were transiently transfected with a control vector (flag) or the indicated yAPCL1257 constructs, fixed on day 3 and stained with an anti-β-catenin antibody. The percentages indicate the number of β-catenin-positive cells (n = 100), and the data are presented as the mean ± standard deviation of three independent experiments. * p<0.003 compared with mutant number 1, Student's t-test. E, SW480 cells were transiently transfected with the indicated yAPCL1257 constructs and N-terminal flag-tagged Axin on day 1. The cells were fixed on day 3 and were stained with an anti-flag antibody and Hoechst dye. The percentages indicate the number of cells without colocalisation (n = 100), and the data are presented as the mean ± standard deviation of three independent experiments. * p<0.009 compared with mutant number 1, Student's t-test. F, Intracellular localisation of the indicated yAPCL1257 constructs transfected as described in (D). Bar, 10 μM.

The immunoprecipitation of yAPCL(1091–1257) followed by western blotting revealed a pattern of three bands (**fig. 6A, B**). Alkaline phosphatase treatment of the immunoprecipitate eliminated the upper band and strongly decreased the intensity of the middle band, thereby indicating that these bands represent phosphorylation events. The lower band, the intensity of which increased upon alkaline phosphatase treatment, therefore corresponded to the unphosphorylated form of yAPCL(1091–1257). To investigate the possibility that the 20R2 might be phosphorylated, we created additional constructs in the context of yAPCL(1091–1257) in which we introduced the L1168V mutation (construct 1) and individually mutated each conserved and potential phosphorylatable Ser/Thr residue within the 20R2 (constructs 2–5) (**fig. 6A**). The L1168V mutation was an unavoidable consequence of the cloning strategy, but it did not influence the phosphorylation pattern (**fig. 6B**). The mutation of Thr1155 to Ala (construct 2) also did not affect the phosphorylation pattern. Each Ser residue was individually mutated on the background of the LV mutation; S1164 (construct 4) was a strong determinant of phosphorylation, and S1160 and S1167 (constructs 3 and 5, respectively) were weaker determinants of phosphorylation (**fig. 6B**), as evidenced by the relative reduction in the intensity of the upper band. The differences between the Ser residues might be explained by the disruption of a phosphorylation site (S1164, construct 4) and the alteration of a kinase recognition site (S1160 and S1167, constructs 3 and 5, respectively). Notably, the intensity of the middle band did not decrease in the mutants, indicating the presence of a phosphorylation site independent of the four conserved Ser/Thr residues.

### The residues of the 20R2 that are involved in phosphorylation are also involved in the down-regulation of β-catenin mediated by truncated APCL and the colocalisation of Axin with truncated APCL

We next analysed the functional consequences of individually mutating each of the four conserved Ser/Thr residues in the context of yAPCL1257-L1168V (**fig. 6C–F**). We preliminary established that the L1168V mutation did not affect the ability of the ectopically expressed protein to inhibit β-catenin transcriptional activity (**fig. S5**). The intracellular localisation of all the constructs was similar (**fig. 6F**). TOP/FOP reporter assays (**fig. 6C**) and β-catenin staining (**fig. 6D** **, S6**) in transiently transfected SW480 cells indicated that each Ser/Thr residue was required to prevent the accumulation of β-catenin and to inhibit its transcriptional activity. Remarkably, the T1155A mutation almost abolished these activities of yAPCL1257. These results qualitatively correlated with the ability of these ectopically expressed proteins to colocalise with Axin (**fig. 6E** **, S7**), as the mutation of each Ser/Thr residue resulted in a partial loss of colocalisation with flag-Axin. One notable quantitative discrepancy occurred with yAPCL1257-L1168V-T1155A, which colocalised with flag-Axin in a greater proportion of cells than expected based on the β-catenin staining and reporter assays. We hypothesised that T1155 might be involved in other functions in addition to contributing to Axin colocalisation. Together, we concluded that the down-regulation of β-catenin mediated by truncated APCL as well as the Axin/APCL colocalisation depended on the presence of the conserved T1155 residue. The dependence on the conserved Ser residues in the 20R2 suggests a phosphorylation-dependent regulation of truncated APCL impacting on β-catenin activity and Axin colocalisation.

### The SAMP repeats of APCL and APC do not compensate for the inactivation of the 20R2 in terms of down-regulating β-catenin

**Figure 7:**
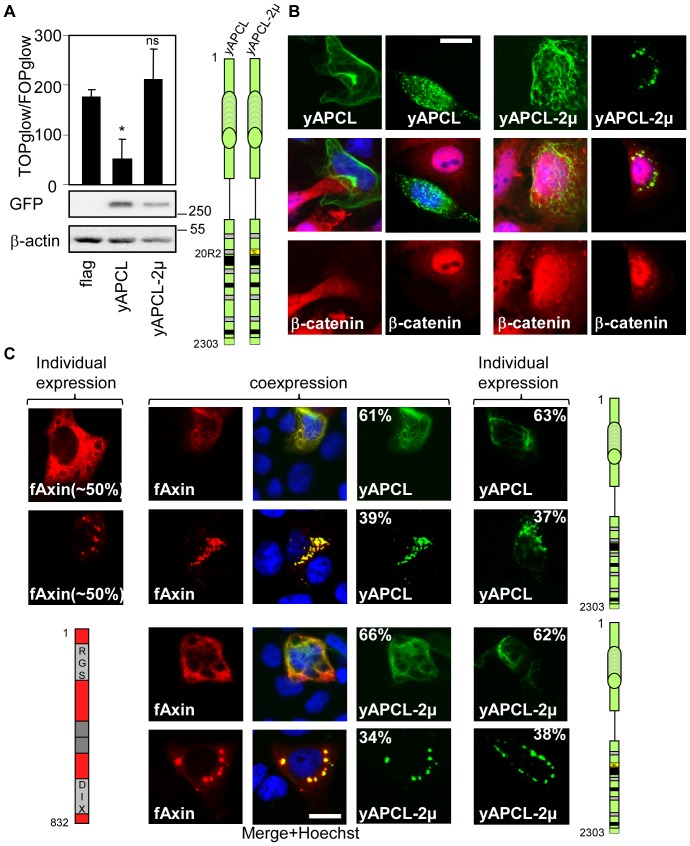
The 20R2 of APCL is required to inhibit β-catenin transcriptional activity (A) and to down-regulate β-catenin (B) but is not necessary to recruit Axin (C) in the presence of SAMP repeats. A, SW480 cells were transiently transfected on day 1 with reporter plasmids and 100(flag) or the indicated APCL constructs. yAPCL-2μ contains the mutations shown in figure 4A. TOP/FOP reporter assays were performed on day 3 to measure β-catenin transcriptional activity. The data are presented as the mean ± standard deviation of three independent values from a representative experiment. * p<0.004 compared with flag, Student's t-test. ns, not significant. In a parallel experiment, cells were transiently transfected with 1 μg of the indicated plasmids on day 1. Cell extracts were prepared on day 3 and were subjected to western blotting using the indicated antibodies. The molecular weights are presented in kDa. B, SW480 cells were transiently transfected with control vector (flag) or the indicated APCL constructs, fixed on day 3 and stained with an anti-β-catenin antibody. Bar, 10 μM. C, DLD1 cells were transiently transfected on day 1 with the indicated APCL constructs or N-terminal flag-tagged Axin, either individually or in combination. The cells were fixed on day 3 and were stained with an anti-flag antibody. Where applicable, the percentages indicate the proportion of different localisation patterns in the transfected cells. The imaging parameters were identical for each type of tag. Bar, 10 μM.

APCL contains two identifiable SAMP repeats that may contribute to β-catenin degradation and compensate for the deleterious effect of mutating the 20R2. Therefore, we extended the yAPCL1257-2μ construct up to position 1357, shortly after the first SAMP repeat (**fig. S8**). This construct did not restore flag-Axin colocalisation (**fig. S8A**) or β-catenin down-regulation (**fig. S8B**). Interestingly, the lack of Axin colocalisation with the first SAMP repeat corresponds with previous results showing that both APCL SAMP repeats are required to interact with Axin [38]. Next, we mutated the 20R2 in the context of full-length yAPCL (yAPCL-2μ, **fig. 7A, B**). This produced a construct that had no effect on β-catenin stability or transcriptional activity (**fig. 7A, B**) but still colocalised with flag-Axin (**fig. 7C**). β-catenin colocalised with yAPCL-2μ in the dotty pattern but not in the fibre-like pattern, which might be related to a low binding affinity, as has been recently suggested [59]. We concluded that the SAMP repeats and the other 20Rs of APCL cannot compensate for the inactivation of the 20R2 in terms of down-regulating β-catenin.

**Figure 8:**
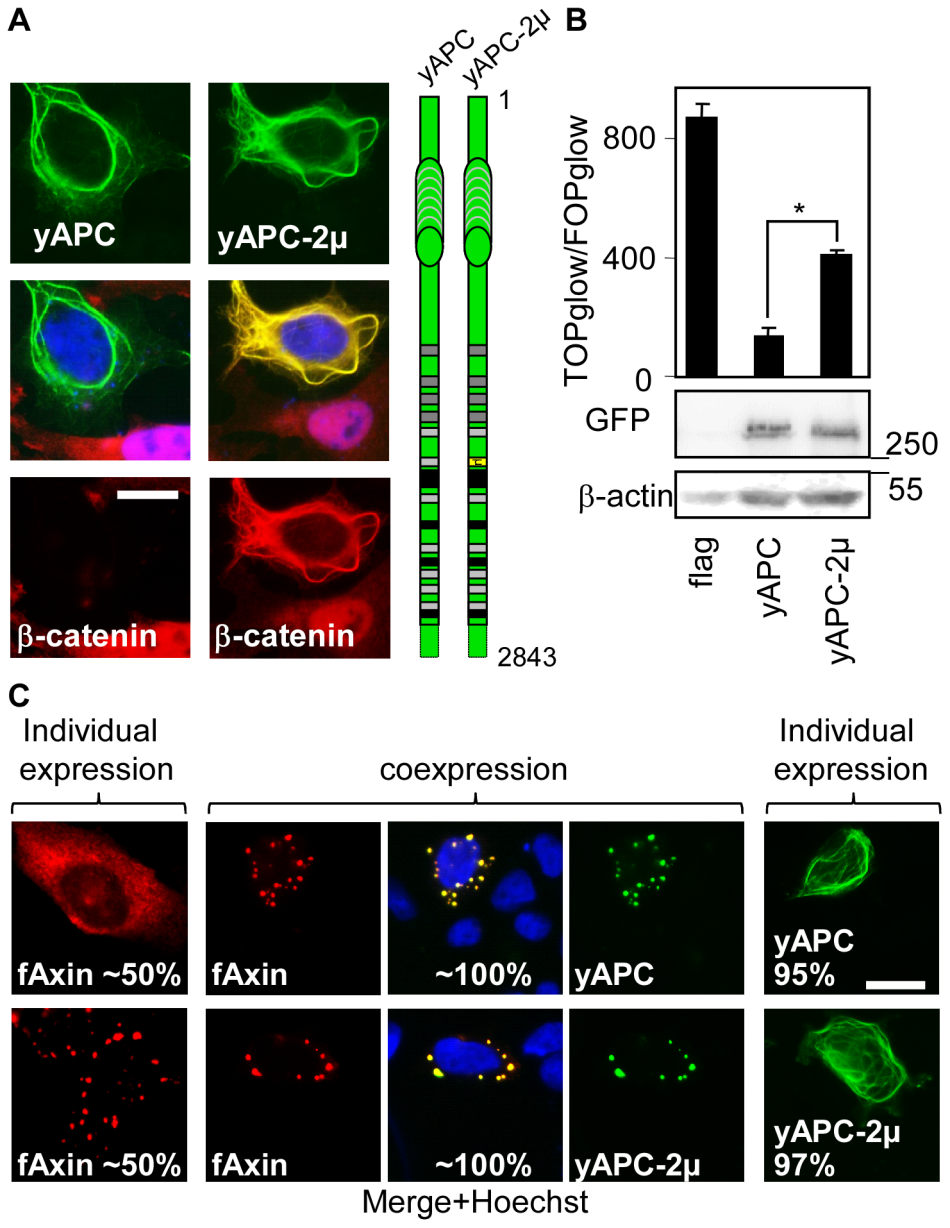
The 20R2 of APC is required to inhibit β-catenin transcriptional activity (A) and to down-regulate β-catenin (B) but was not necessary to recruit Axin in the presence of SAMP repeats (C). A, SW480 cells were transiently transfected with control vector (flag) or the indicated APC constructs, fixed on day 3 and stained with an anti-β-catenin antibody. Bar, 10 μM. C, DLD1 cells were transiently transfected on day 1 with the indicated APC constructs or N-terminal flag-tagged Axin, either individually or in combination. The cells were fixed on day 3 and were stained with an anti-flag antibody. Bar, 10 μM. B, SW480 cells were transiently transfected on day 1 with reporter plasmids and 100 ng of empty vector (flag) or the indicated APC constructs. yAPC-2μ contains the mutations illustrated in figure 4A. TOP/FOP reporter assays were performed on day 3 to measure β-catenin transcriptional activity. The data are presented as the mean ± standard deviation of three independent values from a representative experiment. * p<0.0001 compared with yAPC, Student's t-test. In a parallel experiment, cells were transiently transfected with 1 μg of the indicated plasmids on day 1. Cell extracts were prepared on day 3 and were subjected to western blotting using the indicated antibodies. The molecular weights are presented in kDa. C, DLD1 cells were transiently transfected on day 1 with the indicated APC constructs or N-terminal flag-tagged Axin, either individually or in combination. The cells were fixed on day 3 and were stained with an anti-flag antibody. Where applicable, the percentages indicate the proportion of different localisation patterns in the transfected cells. The imaging parameters were identical for each type of tag. Bar, 10 μM.

APC has three SAMP repeats (**fig. 1**) that bind to Axin, and a role in β-catenin degradation has been demonstrated for the first one [24], [25]. Therefore, the same eight point mutations that inactivated APCL were introduced in the context of yAPC1641 (**fig. 1**) to create yAPC1641-2μ (**fig. S9**). In contrast to yAPC1641, yAPCL1641-2μ did not down-regulate β-catenin (**fig. S9A**) and did not affect β-catenin transcriptional activity when expressed at low levels (**fig. S9B**). Interestingly, yAPC1641-2μ excluded β-catenin from the nucleus (**fig. S9A**). This correlated with the observation that high levels of yAPC1641-2μ inhibited β-catenin transcriptional activity (**fig. S9B**). This might be explained by either sequestration [60], [61] or extremely efficient nuclear export [29] linked to APC. Next, we mutated the 20R2 in the context of full-length yAPC (yAPC-2μ, **fig. 8**). In contrast to full-length wild-type APC, yAPC-2μ was unable to target β-catenin for degradation but did sequester β-catenin outside the nucleus (**fig. 8A**). This second observation likely explains the residual activity measured in the TOP/FOP reporter assay (**fig. 8B**). yAPC-2μ colocalised with flag-Axin (**fig. 8C**). We concluded that the 20R2 of APC was necessary to down-regulate β-catenin and that the SAMP repeats and the other 20Rs of APC cannot compensate for 20R2 inactivation in the down-regulation of β-catenin.

### The first SAMP repeat in APC promotes Axin oligomerisation

**Figure 9:**
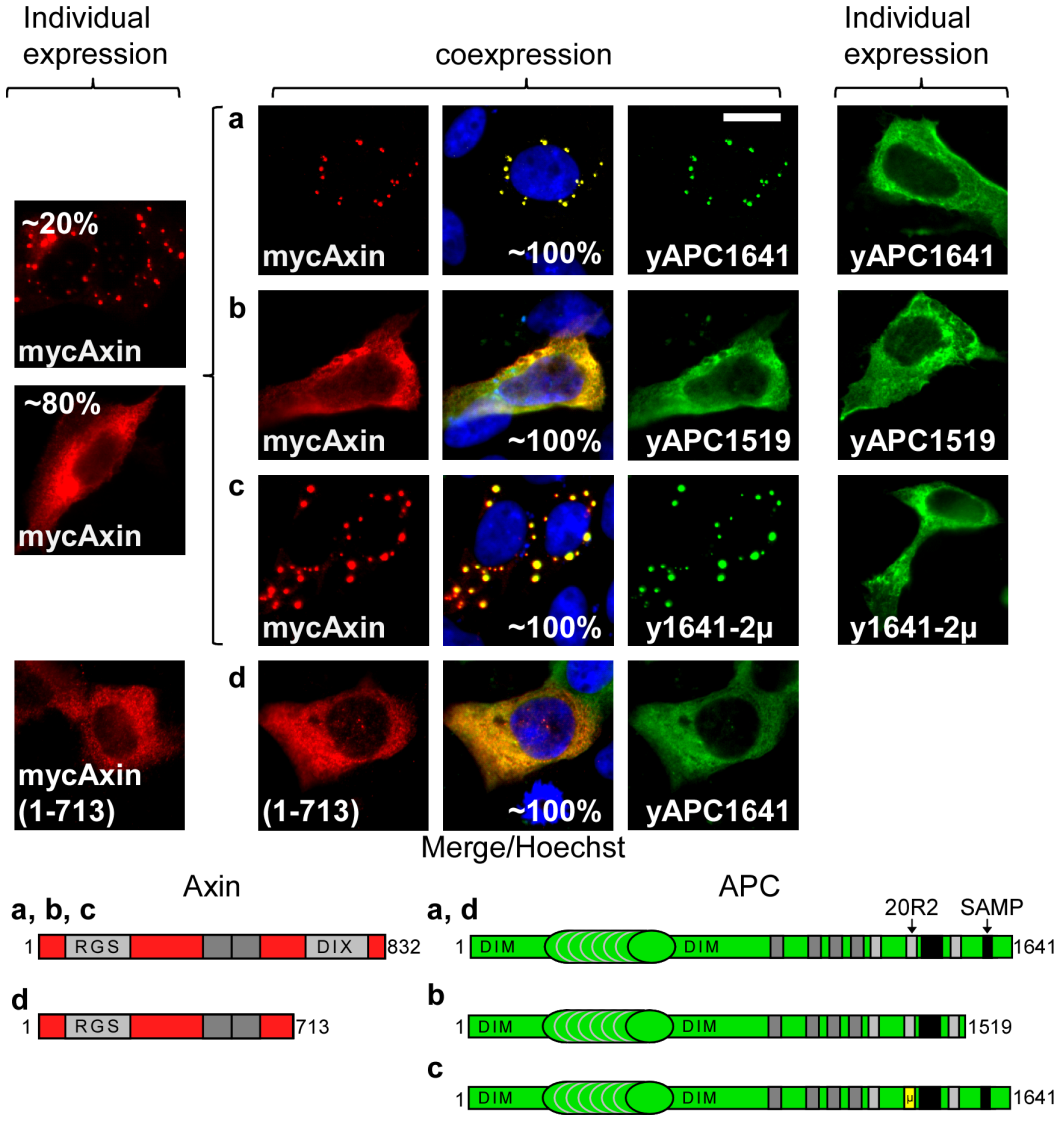
The first SAMP repeat of truncated APC is required for Axin oligomerisation, whereas the 20R2 is not necessary. DLD1 cells were transiently transfected on day 1 with the indicated wild-type or mutant APC constructs and N-terminal myc-tagged Axin, either individually or in combination. yAPC1641-2μ contains the mutations presented in figure 4A. The cells were fixed on day 3 and were stained with an anti-myc or an anti-flag antibody. Where applicable, the percentages indicate the proportion of different localisation patterns in the transfected cells. The imaging parameters were identical for each type of tag. Bar, 10 μM.

To understand the role of the first SAMP repeat in APC, we performed colocalisation experiments in DLD1 cells using the yAPC1641 and myc-Axin constructs (**fig. 9**). yAPC1641 exhibited a diffuse cytoplasmic distribution when expressed alone. However, co-expressing yAPC1641 and myc-Axin resulted in the formation of bright cytoplasmic dots containing yAPC1641 and myc-Axin in nearly all the co-transfected cells. Dot formation depended on the presence of a short domain containing the SAMP repeat in APC (compare yAPC1641 and yAPC1519) and the DIX domain of Axin, which is known to mediate its oligomerisation (compare myc-Axin and myc-Axin(1–713)) [46]. We concluded that truncated APC promoted Axin oligomerisation, likely via the binding of Axin to the SAMP repeat.

### APC-mediated Axin oligomerisation is independent of the 20R2

To investigate the influence of the 20R2 on dot formation, we repeated the colocalisation experiments and compared yAPC1641 with yAPC1641-2μ (**fig. 9**) and yAPC with yAPC-2μ (**fig. 8C** **, S10**). Inactivating the 20R2 did not affect the efficiency of dot formation with Axin. We concluded that APC-stimulated Axin oligomerisation was independent of the 20R2.

### Axin, but not Axin2, stimulates co-oligomerisation with APC, but not APCL

**Figure 10:**
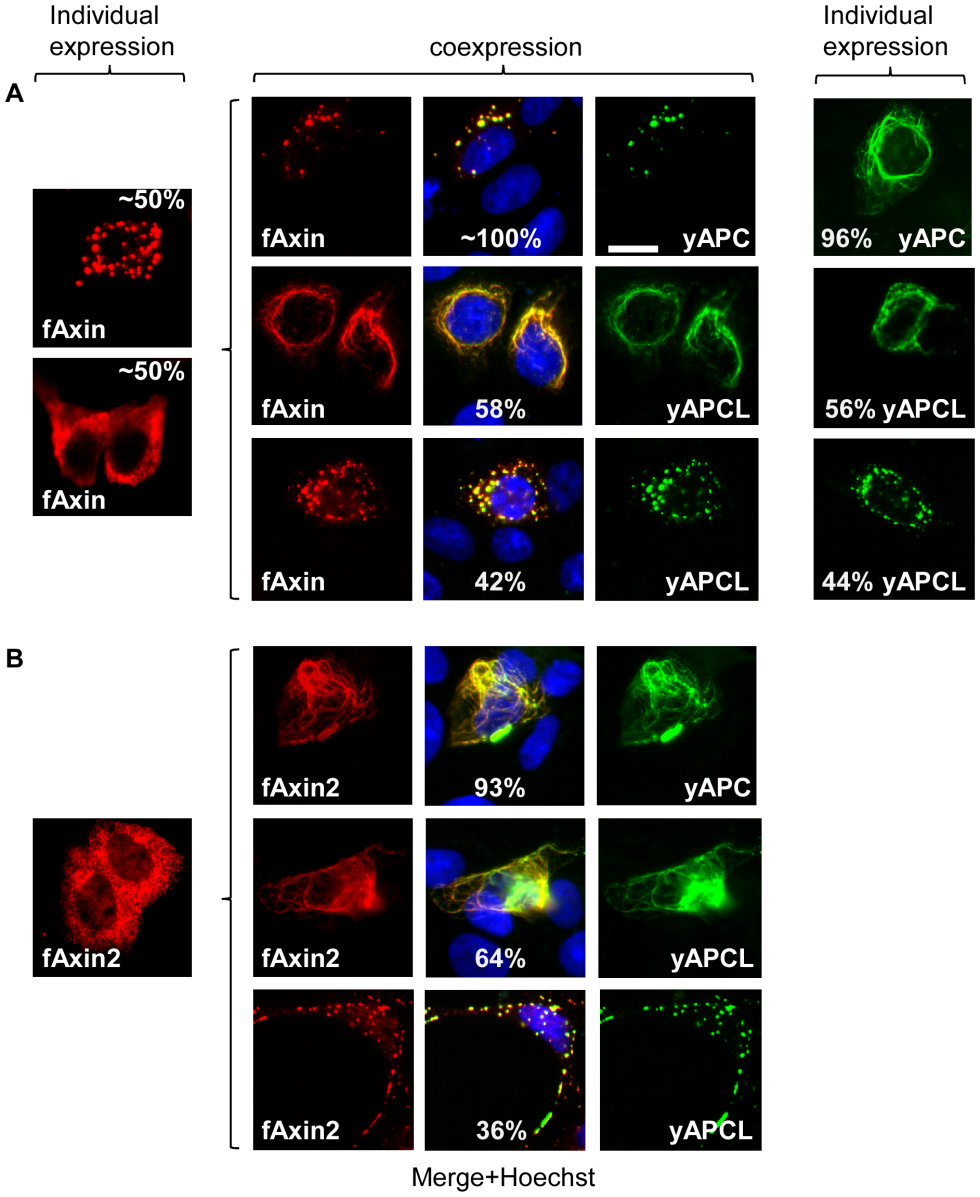
Stimulation of co-oligomerisation occurs only with the Axin/APC pair. DLD1 cells were transiently transfected on day 1 with full-length APC or APCL constructs or N-terminal flag-tagged Axin or Axin2, either individually or in combination. The cells were fixed on day 3 and were stained with an anti-flag antibody. Where applicable, the percentages indicate the proportion of different localisation patterns in the transfected cells. The imaging parameters were identical for the flag tag; the yAPCL signal was more intense than the yAPC signal. Bar, 10 μM.

We discovered that myc-Axin (**fig. 2** **, S10**) and flag-Axin (**fig. 8C**) delocalised yAPC from the cytoskeleton fibres and stimulated the incorporation of yAPC into dots. This effect was not observed with yAPCL (**fig. 2** **,** **7C**). Furthermore, flag-Axin2 was unable to delocalise yAPC or yAPCL from the cytoskeleton (**fig. 10**). Thus, only Axin specifically delocalised APC from the cytoskeleton fibres.

## Discussion

The core destruction complex that targets β-catenin for degradation integrates APC and Axin in a process that catalyses the phosphorylation, ubiquitination and proteolysis of β-catenin. The mechanistic details are not completely understood. The results presented here (summarised in Tables 1 and 2) reveal an unexpected view of the molecular machinery that controls the fate of β-catenin.

**Table 1:** Summary of the colocalisation and β-catenin experiments.

Construct	Colocalisation with:					
	Axin	Axin2	β-catenin	β-catenin degradation		Reduction in TOP/FOP
yAPCL	+			+		+
yAPCL-2μ	+		+	−		−
yAPCL1728	+					
yAPCL1357-2μ	−					
yAPCL1257	+			+		+
yAPCL1257-2μ	−		+	−		−
yAPCL1257-2apc	+			+		+
yAPCL1179	+					
yAPCL1147	−					
yAPCL730	−	+				
yAPC	+			+		+
yAPC-2μ	+			−		+/−
yAPC1641	+			+		+
yAPC1641-2μ	+			−		+/−

**Table 2:** Summary of the co-oligomerisation experiments.

Construct	Stimulated co-oligomerisation with:	
	Axin	Axin2
yAPC	+	−
yAPC-2μ	+	
yAPC1641	+	
yAPC1641-2μ	+	
yAPC1519	−	
yAPCL	−	−

### Role of the 20R2

The 20R2 cannot bind to β-catenin [18], [30]. Rather, we provide evidence that the 20R2 of both APC and APCL interacts with Axin. This conclusion is based on the results of colocalisation experiments using ectopically expressed constructs. Our attempts to strengthen our observations with co-immunoprecipitation experiments were not successful. These interactions possibly occur with low affinity and/or are highly dynamic, similar to the previously described Dishevelled and Axin oligomers [44]–[46]. Meanwhile, the various and minor modifications that we introduced into the 20R2 of different APCL constructs led to relatively important effects on Axin/APCL colocalisation, strongly arguing against the unspecific aggregation of the two proteins. What we interpreted as an Axin/APCL interaction through the 20R2 was observed with ectopically expressed constructs. This interaction awaits confirmation at the endogenous level, which might be difficult due to the two SAMP repeats that also bind to Axin when present together [38]. Similarly, the interaction between Axin and the 20R2 of APC from colon cancer cells (truncated before the SAMP repeats) might be concealed by the presence of an already known Axin-binding site located before the 20R2 [8], [41].

Mutating the 20R2 clearly decreased or eliminated β-catenin down-regulation by APC or APCL, even in the presence of SAMP repeats, which are the classical Axin-binding sites in APC [21]. The 20R2 in Drosophila APC constitutes an important determinant of β-catenin degradation [8]. Thus, the 20R2 is necessary to down-regulate β-catenin, whereas the SAMP repeats are not sufficient to down-regulate β-catenin when the 20R2-dependent interaction with Axin is abolished. A previous publication reported that Axin-mediated β-catenin phosphorylation depends on APC when Axin cannot interact with β-catenin [62]. Another report showed that an internal APC fragment extending from the beginning of the 15R up to the end of the 20R3, but not a fragment interrupted shortly before the 20R2, stimulated β-catenin phosphorylation [47]. In conjunction with our results, these results suggest that β-catenin phosphorylation requires 20R2-bound Axin.

Our results indicate that Axin does not colocalise with either the first or third 20R of APCL and that neither APC nor APCL contain another 20R that could functionally complement 20R2 inactivation. Comparing all the 20R sequences from APC and APCL (**fig. S11**) revealed that 20R7 through 20R12 share the four residues that we identified as being important for Axin binding and β-catenin degradation. Moreover, the 20R1 of APC is very similar to the 20R2, suggesting that most of the specificity determinants for Axin binding are provided by sequences outside the original short boundaries of the 20R2. Indeed, a comparison after extending in the N- and C-terminal directions revealed that the extended regions were more conserved in the 20R2s of APC and APCL than in any other homologous 20R pair. The stronger selective pressure on the 20R2 is likely related to the conservation of the elements that create a specific Axin-binding site.

### Role of the SAMP repeats

The SAMP repeats of APC cannot complement 20R2 inactivation, but they are important for inhibiting tumour development [63], [64] and are crucial for β-catenin degradation under specific conditions (see below) [24], [25]. Our results indicate that a short domain containing the first SAMP repeat in APC is necessary to initiate Axin recruitment into cytoplasmic dots. This recruitment also requires the DIX domain of Axin, which mediates Axin oligomerisation [44], [46]. The SAMP repeat binds to the RGS domain of Axin [21]. We found that myc-Axin exhibits diffuse cytoplasmic localisation in most of the transfected cells when it is expressed alone, but removing the N-terminal region containing the RGS domain elicits dot formation, which we interpret as oligomerisation because the formation of this pattern requires the DIX domain. These data suggest that the RGS domain inhibits Axin oligomerisation. It follows that one role of the SAMP repeat may be to release the inhibitory constraint exerted by the RGS on Axin oligomerisation. Our results correspond with a recent report demonstrating that stably transfected full-length APC promotes Axin oligomerisation, an event that does not occur when the RGS domain contains point mutations that abolish the interaction with the SAMP repeats [45]. Thus, the role of the SAMP repeat is intimately linked to Axin oligomerisation. It was reported [43] that ectopically expressed Axin lacking the DIX domain was fully competent at catalysing β-catenin phosphorylation, but the subsequent degradation of β-catenin was blocked. Thus, Axin oligomerisation correlates with the degradation of phospho-β-catenin. Therefore, we propose that Axin oligomerisation promoted by the RGS-SAMP interaction might be necessary to catalyse the degradation of phospho-β-catenin under endogenous conditions. In line, another report mentioned that Axin lacking its RGS domain, and therefore more prone to spontaneous oligomerisation, was more active at targeting β-catenin for degradation than full-length Axin [41].

### A new model for the destruction complex

**Figure 11:**
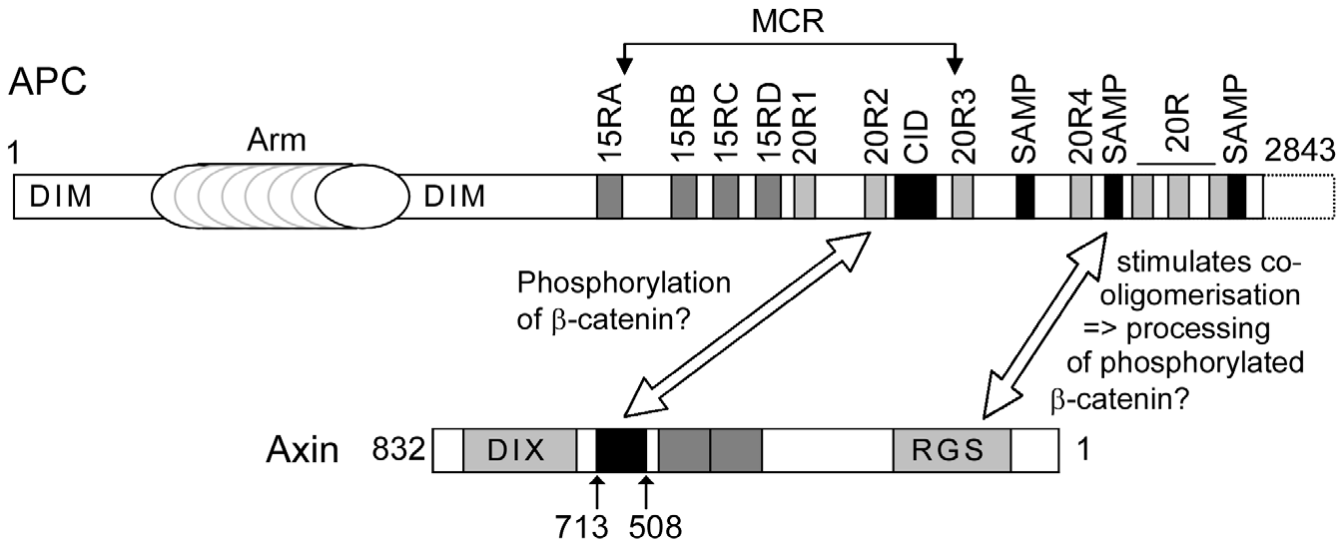
Working model. The interactions between the 20R2 of APC and amino acids 508-713 of Axin and between the SAMP repeat of APC and the RGS domain of Axin are shown with double arrows. We attribute putative functions to these interactions. See figure 1 for details.

The recognition of two different types of Axin binding sites on APC that are apparently associated with two different functions suggests a new model for the destruction complex (**fig. 11**). Accordingly, Axin bound to the 20R2 catalyses β-catenin phosphorylation, whereas Axin bound to the SAMP repeat catalyses the subsequent degradation of phosphorylated β-catenin by promoting Axin oligomerisation.

This model may help explain previously published and apparently contradictory data. On the one hand, immunoprecipitation experiments using an antibody against Axin have shown that the β-catenin destruction complex remains essentially intact upon Wnt stimulation (9). On the other hand, using an antibody against APC, it has been observed that Wnt induces the partial dissociation of APC and Axin [48], [49]. The discrepancies between the two reports might be explained by the disruption of only one type of APC-Axin interaction, either the 20R2-Axin interaction or the SAMP-Axin interaction, which might have been revealed with an APC antibody but not an Axin antibody. The observations that Wnt stimulation inhibits β-catenin degradation without affecting phosphorylation [9] and that an internal APC fragment lacking the SAMP repeats but retaining the 20R2 stimulates β-catenin phosphorylation [47] are consistent with the hypothesis that the partial dissociation of the APC-Axin complex in response to Wnt corresponds with the specific disruption of the RGS-SAMP interaction. Alternatively, contradictory data indicate that Wnt stimulation inhibits β-catenin phosphorylation but not its subsequent processing [65], suggesting a partial disruption of the Axin/APC complex at the level of the 20R2 but not the SAMP repeats.

### The mechanism of action of truncated APC

The SAMP repeats are not necessary for targeting β-catenin for degradation when truncated APC retaining the CiD is ectopically expressed [20]. This raises the question of how the degradation of phospho-β-catenin is achieved under these circumstances. Several observations suggest that the SAMP repeats cooperate with the 15R. First, the SAMP repeats are essential for β-catenin degradation when β-catenin binding to the 15Rs has been abolished either by deletion [25] or the introduction of point mutations [24]. Second, ectopic expression experiments demonstrated that the loss of the 15R was offset by the presence of a SAMP repeat but not by the presence of the 20R1 and/or the 20R3, and vice versa [24]. Third, it has been shown that phospho-β-catenin prefers to bind the 15R rather than the 20R1 [59]. Together, these observations suggest that both the 15R and the SAMP repeats of APC collaborate in the same function. It follows that the 15R may contribute to the degradation of phosphorylated β-catenin and may provide the partial activity of truncated APC in colon cancer cells, as has been discussed recently [59].

### APCL remains wild-type in colon cancer cells

Our data provide a hint at the solution for another conflicting situation in colon cancer cells. β-catenin is stabilised as a consequence of mutations that truncate APC before the first SAMP repeat, despite the expression of APCL [38], [39], [59], Axin and Axin2 [66]. Several functional differences between APC and APCL were recently described [59]. In the present study, we add to these differences by reporting that Axin and APC mutually stimulate co-oligomerisation in punctuate structures (**fig. 10**), which have been proposed by others to represent fully active β-catenin destruction complexes [43], [45], [46], whereas APCL recruits Axin and Axin2 to the cytoskeleton. The origin of this difference is unclear, but the data suggest that APCL differs profoundly from APC in the way it interacts with Axin through the SAMP repeats; this different interaction mode renders APCL an inefficient partner for the degradation of phospho-β-catenin. This may explain why APCL does not need to be mutated in colon cancer. Notably, the ectopic expression of APCL leads to β-catenin degradation. However, this is apparently related to the dependency of APCL on endogenous truncated APC to provide the 15R [59].

As a conclusion, the data presented here and elsewhere converge on a working model that attributes β-catenin phosphorylation to Axin bound to the 20R2 of either APC or APCL and the degradation of phosphorylated β-catenin to the oligomerisation of Axin bound to the SAMP repeats of APC.

## Material and Methods

### Cells

Human embryonic kidney cells expressing SV40 large T antigen (HEK293T) and SW480 and DLD1 colorectal cancer cell lines were obtained from American Type Culture Collection (ATCC) and were maintained in DMEM (PAA Laboratories, Cölbe, Germany) supplemented with 10% foetal calf serum (Perbio Laboratories, Frankfurt am Main, Germany), 1% penicillin and 1% streptomycin (PAA Laboratories). The APC mutations in the cell lines are described in [28], and the resulting truncated APC protein products are reported in [67].

### Antibodies

The following primary antibodies were used in this study: anti-GFP (Roche, Mannheim, Germany), anti-β-actin (Santa Cruz Biotechnologies, Heidelberg, Germany), sheep anti-TGN46 (Serotec, Düsseldorf, Germany), mouse-anti-GM130 (BD Transduction Laboratories, Heidelberg, Germany), rabbit anti-Giantin (Covance, Munich, Germany), rabbit anti-flag and anti-β-catenin H102 (Sigma-Aldrich, Taufkirchen, Germany) and anti-myc (Cell Signaling, Danvers, MA, USA). Secondary antibodies coupled to horseradish peroxidase or Cy3 were from Dianova, Hamburg, Germany.

### Plasmids

The plasmids expressing the YFP-APC and YFP-APCL fusion proteins were constructed using standard molecular biology methods. The plasmid sequences are available upon request. pcDNAflag [21] was used as a control vector. Human APCL was obtained from H. Nakagawa and Y. Nakamura [37]. N-terminal myc-tagged rat Axin and mutants thereof were kind gifts from T. Kadoya and A. Kikuchi [68]. N-terminal flag-tagged mouse Axin was from H. Clevers, and N-terminal flag-tagged mouse Axin2 has been described elsewhere [21].

### Plasmid transfection

Plasmids were transfected into cells overnight using 5 μl of polyethylenimine (1 mg/ml) per μg of DNA. For DLD1 and SW480 cells, 2 μg of total plasmid DNA was transiently transfected into 250,000 cells in 35 mm dishes. For HEK293T cells, 10 μg of total plasmid DNA was transiently transfected into 5,000,000 cells in 85 mm dishes.

### TOP/FOP reporter assays [69]

The TOPglow reporter consists of a tandem repeat of four TCF/LEF1 binding sites inserted in front of a TATA box [70] that drive luciferase expression in a β-catenin-dependent manner. In the FOPglow reporter, the four binding sites are mutated to abolish TCF/LEF1 binding. pUHD16.1, which expresses β-galactosidase, was used as an internal control to correct for variations in transfection efficiency and was transiently transfected with the FOPglow or TOPglow plasmids at an equimolar ratio (300 ng each). The transcriptional activity, which was measured 48 h post-transfection, was defined as the ratio of the TOPglow to the FOPglow luciferase values corrected by the β-galactosidase values.

### Cell extracts

Triton-X100 extracts were prepared in a 20 mM Tris-HCl, pH 7.4, buffer containing 150 mM NaCl, 5 mM EDTA, 1% Triton-X100, 1 mM DTT and 1 mM PMSF.

### Immunoprecipitations

Immunoprecipitations were performed using Triton-X100 extracts containing 50 mM NaF. The immunoprecipitates were washed in a 50 mM Tris-HCl, pH 8, buffer containing 150 mM NaCl, 5 mM EDTA and 1% Triton-X100.

### Western blotting

The blots were developed using Western Lightning™ chemiluminescence reagents (Perkin Elmer Life Sciences, Boston, MA), and the signals were detected using a LAS-3000-Fuji camera (Raytest, Straubenhardt, Germany).

### Immunofluorescence

Immunofluorescence was performed as previously described [71].

## Supporting Information

Figure S1 **The intracellular localisation of Axin is either diffuse or dotty.** DLD1 and SW480 cells were transiently transfected on day 1 with N-terminal tagged expression constructs (mouse flag-Axin, rat myc-Axin or human YFP-Axin). The cells were fixed on day 3 and were stained with an anti-flag or an anti-myc antibody. Bar, 10 μM. **A**, Representative images of Axin intracellular localisation. The imaging parameters were identical for each type of tag. **B**, Quantification of the proportion of cells exhibiting a diffuse versus a dotty pattern. The percentages indicate the number of cells with dots (n = 100), and the data are presented as the mean ± standard deviation of three independent experiments.
(PDF)

Figure S2 **The APCL dots are not Golgi vesicles.** SW480 cells were transiently transfected on day 1 with yAPCL1728. The cells were fixed on day 3 and were stained with the indicated antibodies. Bar, 10 μM.
(PDF)

Figure S3 **Comparison of the 20R2 sequences in APC and APCL from different species.** Residues common to all 20R2 sequences are highlighted in red.
(PDF)

Figure S4 **The 20R2 from a short internal APCL fragment is required to inhibit Axin oligomerisation.** DLD1 cells were transiently transfected on day 1 with the N-terminal YFP-labelled APCL constructs or N-terminal flag-tagged Axin, either individually or in combination. The cells were fixed on day 3 and were stained with an anti-flag antibody and Hoechst dye. Where applicable, the percentages indicate the proportion of different localisation patterns observed in the transfected cells. The imaging parameters were identical for each type of tag. Bar, 10 μM.
(PDF)

Figure S5 **The L1168V mutation in the 20R2 does not affect the ability of truncated APCL to inhibit β-catenin transcriptional activity.** SW480 cells were transiently transfected on day 1 with reporter plasmids and 100 ng of an empty vector (flag) or the indicated N-terminal YFP-tagged APCL constructs. TOP/FOP reporter assays were performed on day 3 to measure β-catenin transcriptional activity (see Material and Methods). The data are presented as the mean ± standard deviation of three independent values from a representative experiment. In a parallel experiment, the cells were transiently transfected with 1 μg of the indicated plasmids on day 1. Cell extracts were prepared on day 3 and were subjected to western blotting using the indicated antibodies.
(PDF)

Figure S6 **Representative cells with or without β-catenin down-regulation upon expression of wild-type or mutant yAPCL1257-LV to illustrate the results presented in** **figure 6C** **.** SW480 cells were transiently transfected with the indicated APCL constructs, fixed on day 3 and stained with an anti-β-catenin antibody and Hoechst dye. A schematic of the mutants is presented in figure 6A. The imaging parameters were identical for each type of tag and antibody. Bar, 10 μM.
(PDF)

Figure S7 **Representative cells with or without Axin colocalisation upon co-expression of wild-type or mutant yAPCL1257-LV to illustrate the results presented in** **figure 6D** **.** SW480 cells were transiently transfected on day 1 with the indicated yAPCL1257 constructs and N-terminal flag-tagged Axin. A schematic of the yAPCL1257 mutants is presented in figure 6A. The cells were fixed on day 3 and were stained with an anti-flag antibody and Hoechst dye. The imaging parameters were identical for each type of tag. Bar, 10 μM.
(PDF)

Figure S8 **The first SAMP repeat of APCL restores neither Axin colocalisation** (**A**)**or β-catenin degradation (B) after mutation of the 20R2.** **A**, DLD1 cells were transiently transfected on day 1 with the indicated N-terminal YFP-labelled APCL constructs or N-terminal flag-tagged Axin, either individually or in combination. The cells were fixed on day 3 and were stained with an anti-flag antibody and Hoechst dye. **B**, SW480 cells were transiently transfected with the indicated APCL constructs, fixed on day 3 and stained with an anti-β-catenin antibody and Hoechst dye. The imaging parameters were identical for each type of tag. Bar, 10 μM.
(PDF)

Figure S9 **The 20R2 of APC truncated after the first SAMP repeat is required to target β-catenin for degradation (A) and to inhibit its transcriptional activity (B).** **A**, SW480 cells were transiently transfected with a control vector (flag) or the indicated APCL constructs, fixed on day 3 and stained with an anti-β-catenin antibody and Hoechst dye. **B**, SW480 cells were transiently transfected on day 1 with reporter plasmids and 100 ng of an empty vector (flag) or the indicated N-terminal YFP-tagged APC constructs (see **fig. 1**, **3A**). TOP/FOP reporter assays were performed on day 3 to measure β-catenin transcriptional activity (see Material and Methods). The data are presented as the mean ± standard deviation of three independent values from a representative experiment. In a parallel experiment, cells were transiently transfected with 1 μg of the indicated plasmids on day 1. Cell extracts were prepared on day 3 and were subjected to western blotting using the indicated antibodies. yAPC1641-2μ contains the mutations shown in figure 5A. Bar, 10 μM.
(PDF)

Figure S10 **The 20R2 of full-length APC is not necessary to promote Axin co-oligomerisation.** DLD1 cells were transiently transfected on day 1 with the indicated N-terminal YFP-tagged APC constructs or N-terminal myc-tagged Axin, either individually or in combination. The cells were fixed on day 3 and were stained with an anti-myc antibody and Hoechst dye. yAPC-2μ contains the mutations shown in figure 5A. Where applicable, the percentages indicate the proportion of different localisation patterns observed in the transfected cells. The imaging parameters were identical for each type of tag. Bar, 10 μM.
(PDF)

Figure S11 **Pairwise comparison of the 20Rs of APC and APCL.** Red indicates amino acids common to the 20R2s of APC and APCL from various species (**fig. S3**), red and blue represent the residues common to the 20R2s of human APC and APCL and the bold black letters indicate the amino acids common within each homologous 20R pair. The asterisks indicate residues from the 20R2 of APCL that are important for Axin binding and β-catenin degradation. Bar, 10 μM.
(PDF)
