# Supplementary figures and images for: Different Roles for the Axin Interactions with the SAMP versus the Second Twenty Amino Acid Repeat of Adenomatous Polyposis Coli
Source: PLoS One. 2014 Apr 10;9(4):e94413. doi: 10.1371/journal.pone.0094413 (PMC3983206; doi:10.1371/journal.pone.0094413)

**Figure S1**

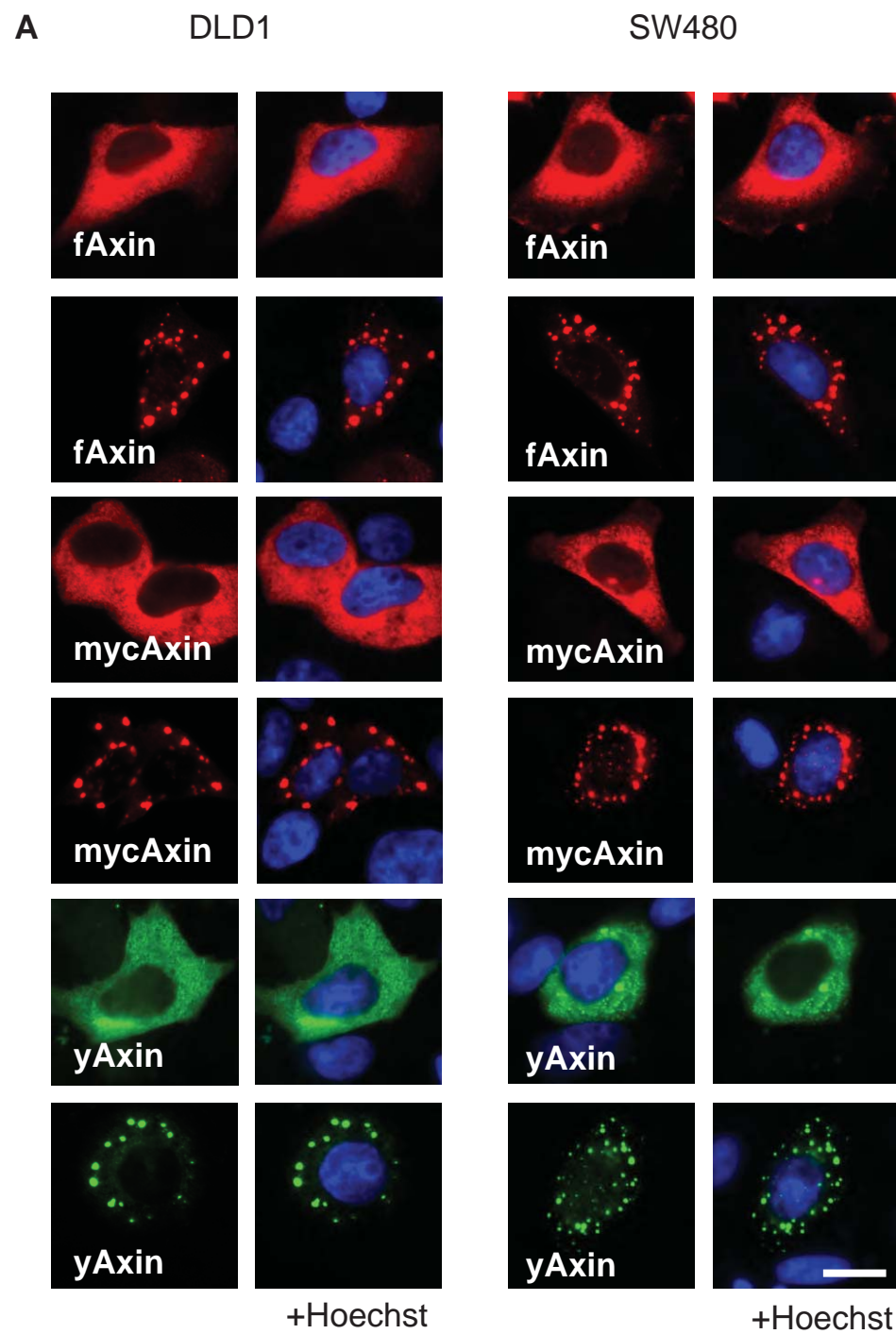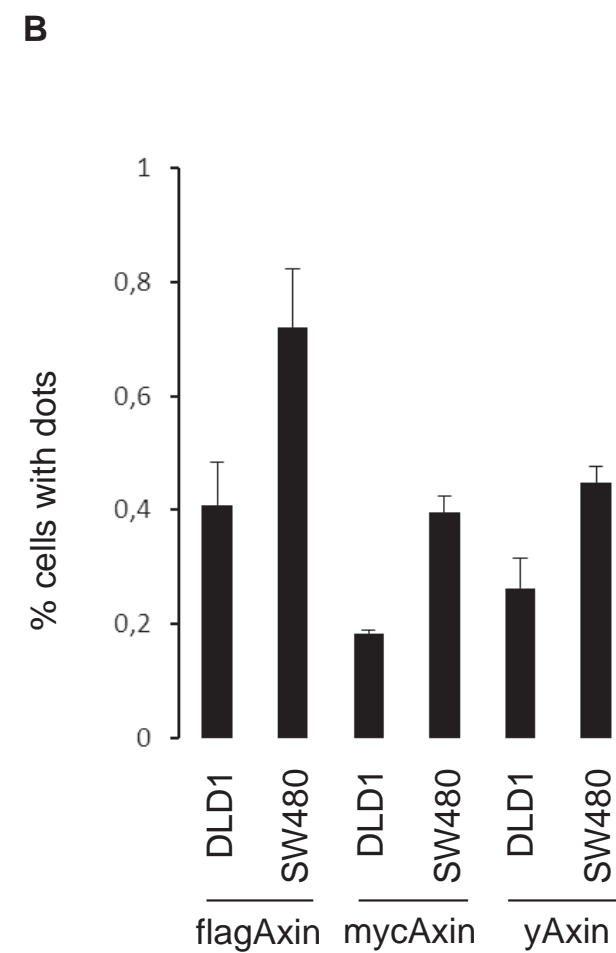

Supplement: Figure S1 — The intracellular localisation of Axin is either diffuse or dotty. DLD1 and SW480 cells were transiently transfected on day 1 with N-terminal tagged expression constructs (mouse flag-Axin, rat myc-Axin or human YFP-Axin). The cells were fixed on day 3 and were stained with an anti-flag or an anti-myc antibody. Bar, 10 μM. A, Representative images of Axin intracellular localisation. The imaging parameters were identical for each type of tag. B, Quantification of the proportion of cells exhibiting a diffuse versus a dotty pattern. The percentages indicate the number of cells with dots (n = 100), and the data are presented as the mean ± standard deviation of three independent experiments. (PDF) [file pone.0094413.s001.pdf]

Figure S2.

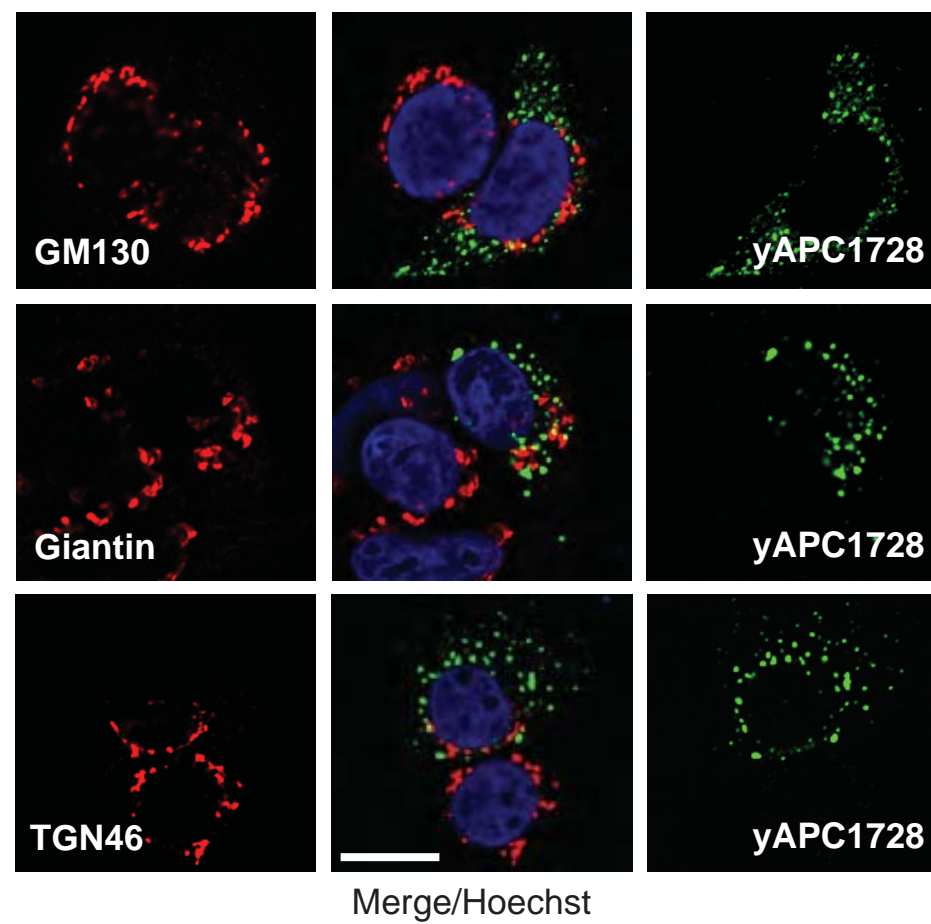

Supplement: Figure S2 — The APCL dots are not Golgi vesicles. SW480 cells were transiently transfected on day 1 with yAPCL1728. The cells were fixed on day 3 and were stained with the indicated antibodies. Bar, 10 μM. (PDF) [file pone.0094413.s002.pdf]

**Figure S5.**

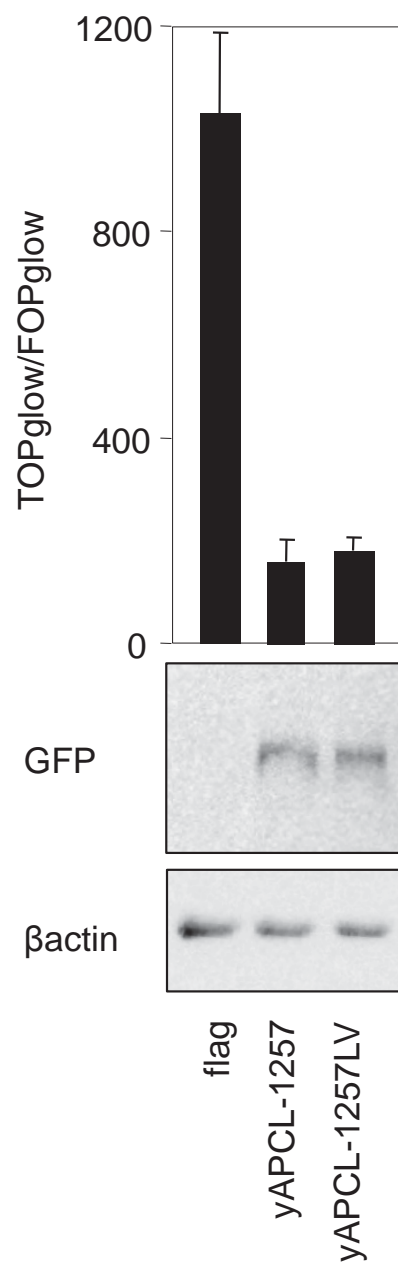

Supplement: Figure S5 — The L1168V mutation in the 20R2 does not affect the ability of truncated APCL to inhibit β-catenin transcriptional activity. SW480 cells were transiently transfected on day 1 with reporter plasmids and 100 ng of an empty vector (flag) or the indicated N-terminal YFP-tagged APCL constructs. TOP/FOP reporter assays were performed on day 3 to measure β-catenin transcriptional activity (see Material and Methods). The data are presented as the mean ± standard deviation of three independent values from a representative experiment. In a parallel experiment, the cells were transiently transfected with 1 μg of the indicated plasmids on day 1. Cell extracts were prepared on day 3 and were subjected to western blotting using the indicated antibodies. (PDF) [file pone.0094413.s005.pdf]

**Figure S6**

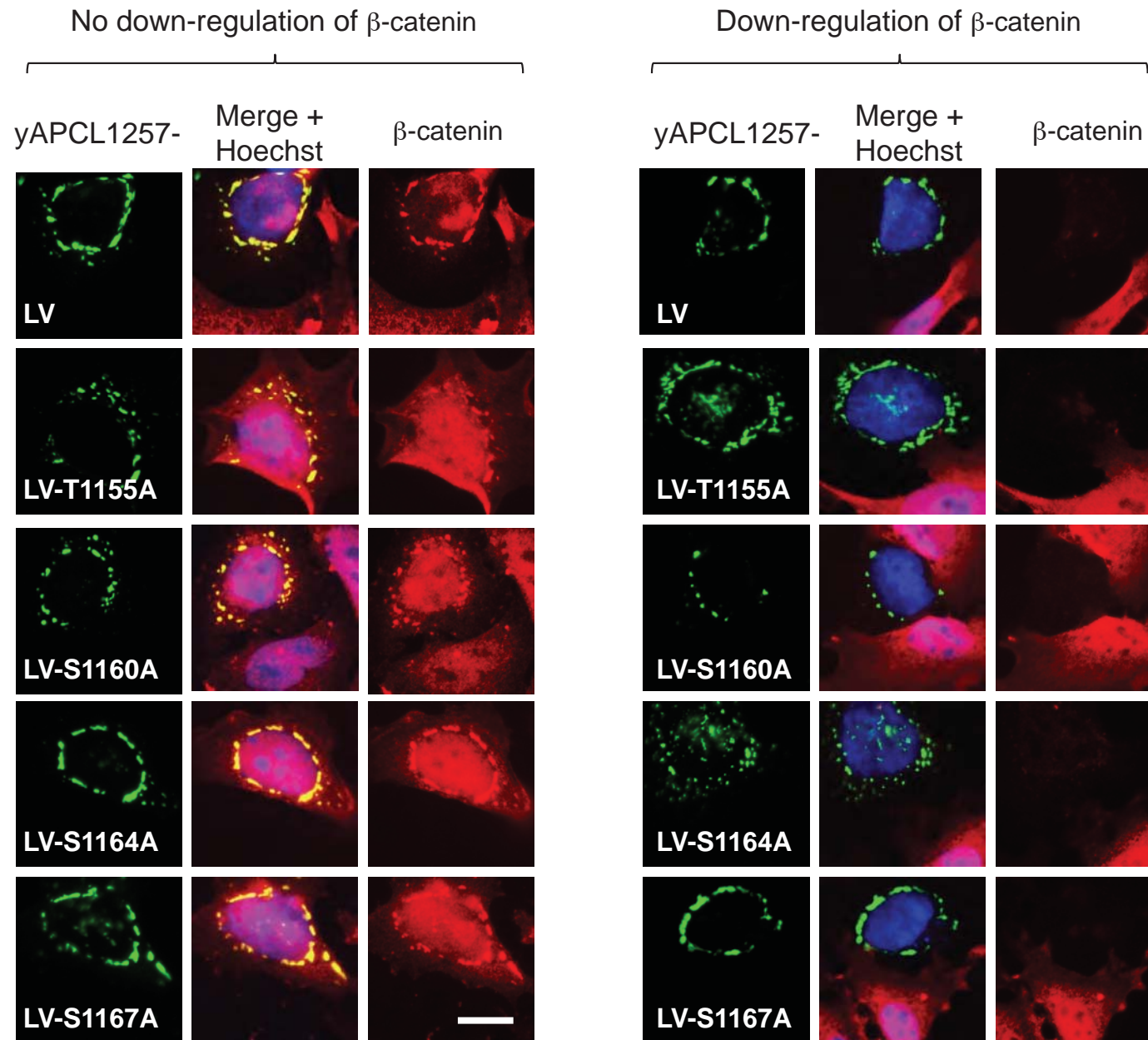

Supplement: Figure S6 — Representative cells with or without β-catenin down-regulation upon expression of wild-type or mutant yAPCL1257-LV to illustrate the results presented in figure 6C . SW480 cells were transiently transfected with the indicated APCL constructs, fixed on day 3 and stained with an anti-β-catenin antibody and Hoechst dye. A schematic of the mutants is presented in figure 6A. The imaging parameters were identical for each type of tag and antibody. Bar, 10 μM. (PDF) [file pone.0094413.s006.pdf]

Figure S7

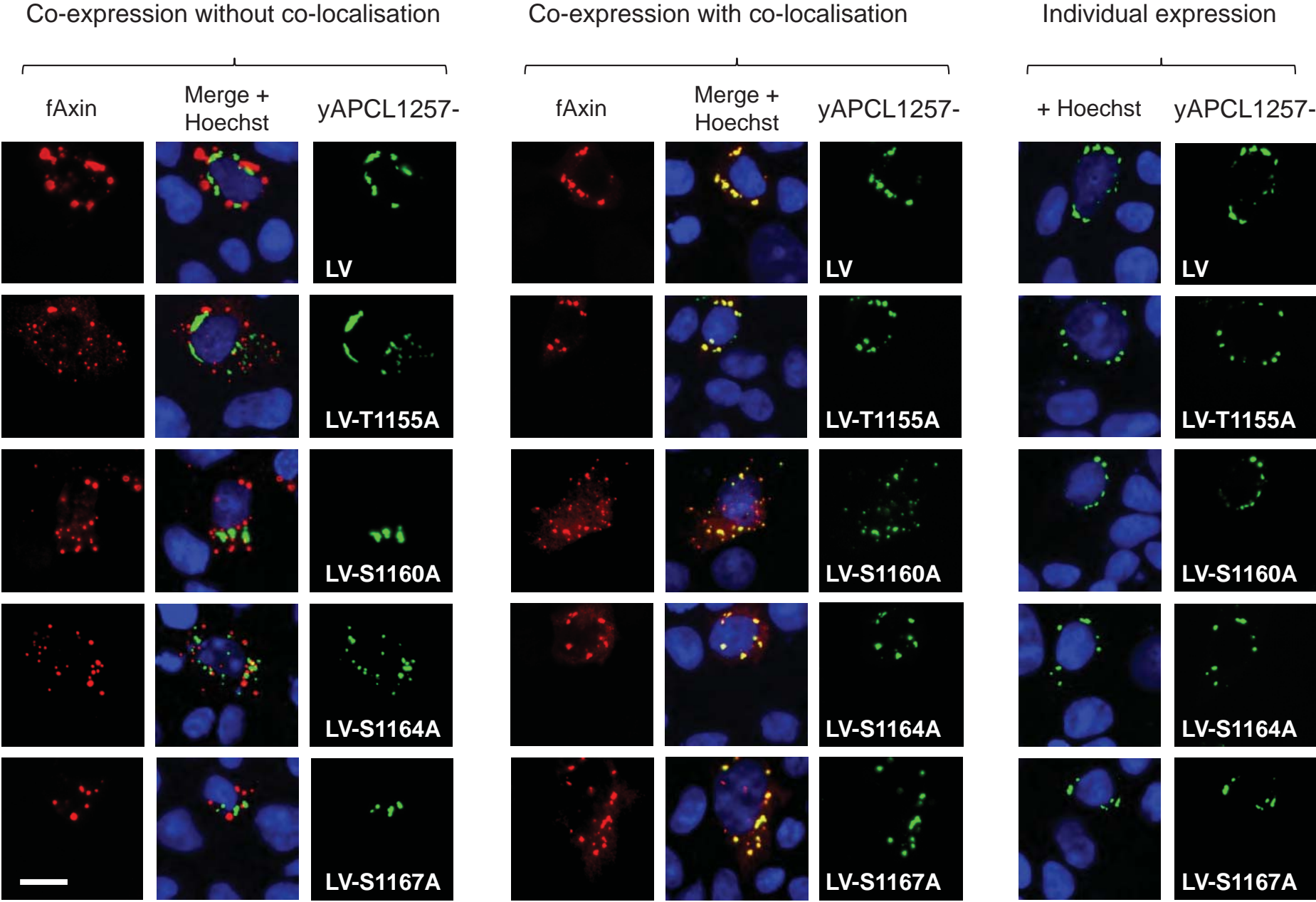

Supplement: Figure S7 — Representative cells with or without Axin colocalisation upon co-expression of wild-type or mutant yAPCL1257-LV to illustrate the results presented in figure 6D . SW480 cells were transiently transfected on day 1 with the indicated yAPCL1257 constructs and N-terminal flag-tagged Axin. A schematic of the yAPCL1257 mutants is presented in figure 6A. The cells were fixed on day 3 and were stained with an anti-flag antibody and Hoechst dye. The imaging parameters were identical for each type of tag. Bar, 10 μM. (PDF) [file pone.0094413.s007.pdf]

Figure S8

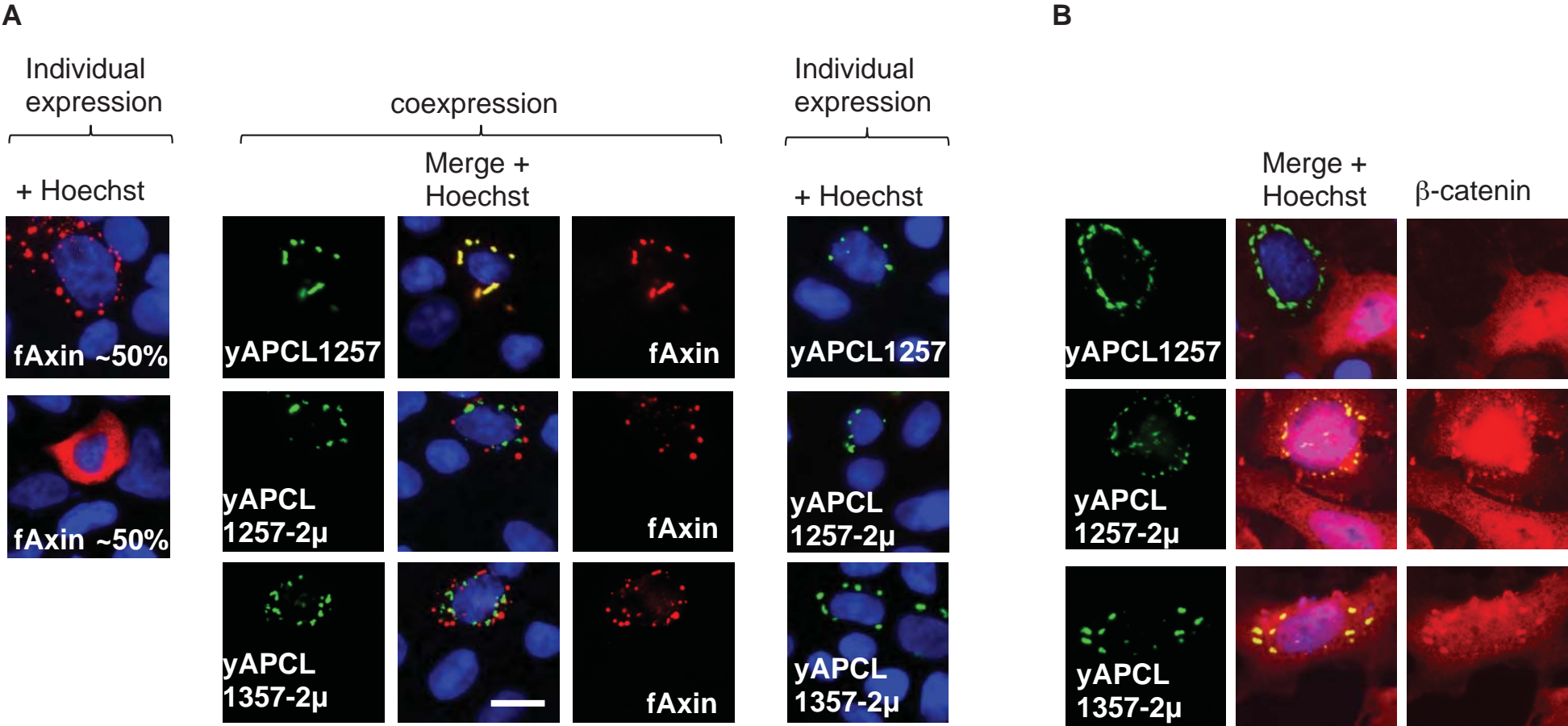

Supplement: Figure S8 — The first SAMP repeat of APCL restores neither Axin colocalisation (A)or β-catenin degradation (B) after mutation of the 20R2. A, DLD1 cells were transiently transfected on day 1 with the indicated N-terminal YFP-labelled APCL constructs or N-terminal flag-tagged Axin, either individually or in combination. The cells were fixed on day 3 and were stained with an anti-flag antibody and Hoechst dye. B, SW480 cells were transiently transfected with the indicated APCL constructs, fixed on day 3 and stained with an anti-β-catenin antibody and Hoechst dye. The imaging parameters were identical for each type of tag. Bar, 10 μM. (PDF) [file pone.0094413.s008.pdf]

Figure S9

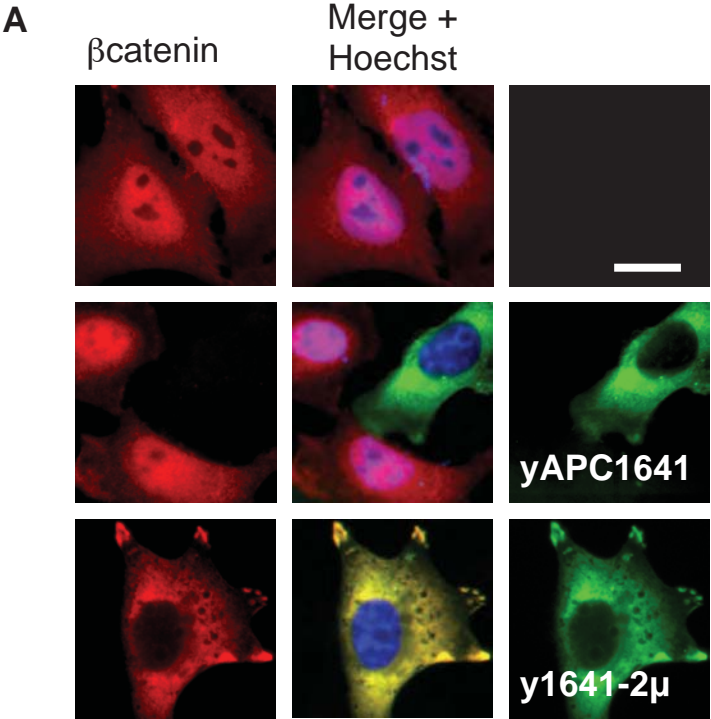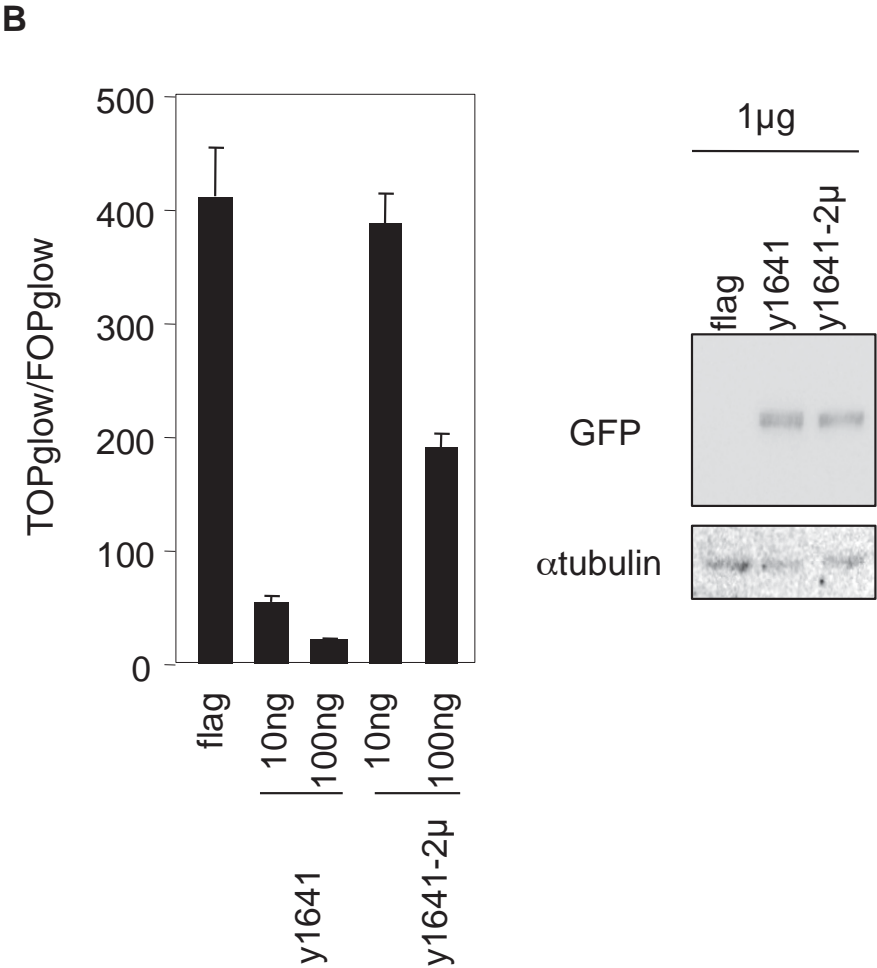

Supplement: Figure S9 — The 20R2 of APC truncated after the first SAMP repeat is required to target β-catenin for degradation (A) and to inhibit its transcriptional activity (B). A, SW480 cells were transiently transfected with a control vector (flag) or the indicated APCL constructs, fixed on day 3 and stained with an anti-β-catenin antibody and Hoechst dye. B, SW480 cells were transiently transfected on day 1 with reporter plasmids and 100 ng of an empty vector (flag) or the indicated N-terminal YFP-tagged APC constructs (see fig. 1 , 3A ). TOP/FOP reporter assays were performed on day 3 to measure β-catenin transcriptional activity (see Material and Methods). The data are presented as the mean ± standard deviation of three independent values from a representative experiment. In a parallel experiment, cells were transiently transfected with 1 μg of the indicated plasmids on day 1. Cell extracts were prepared on day 3 and were subjected to western blotting using the indicated antibodies. yAPC1641-2μ contains the mutations shown in figure 5A. Bar, 10 μM. (PDF) [file pone.0094413.s009.pdf]

**Figure S10**

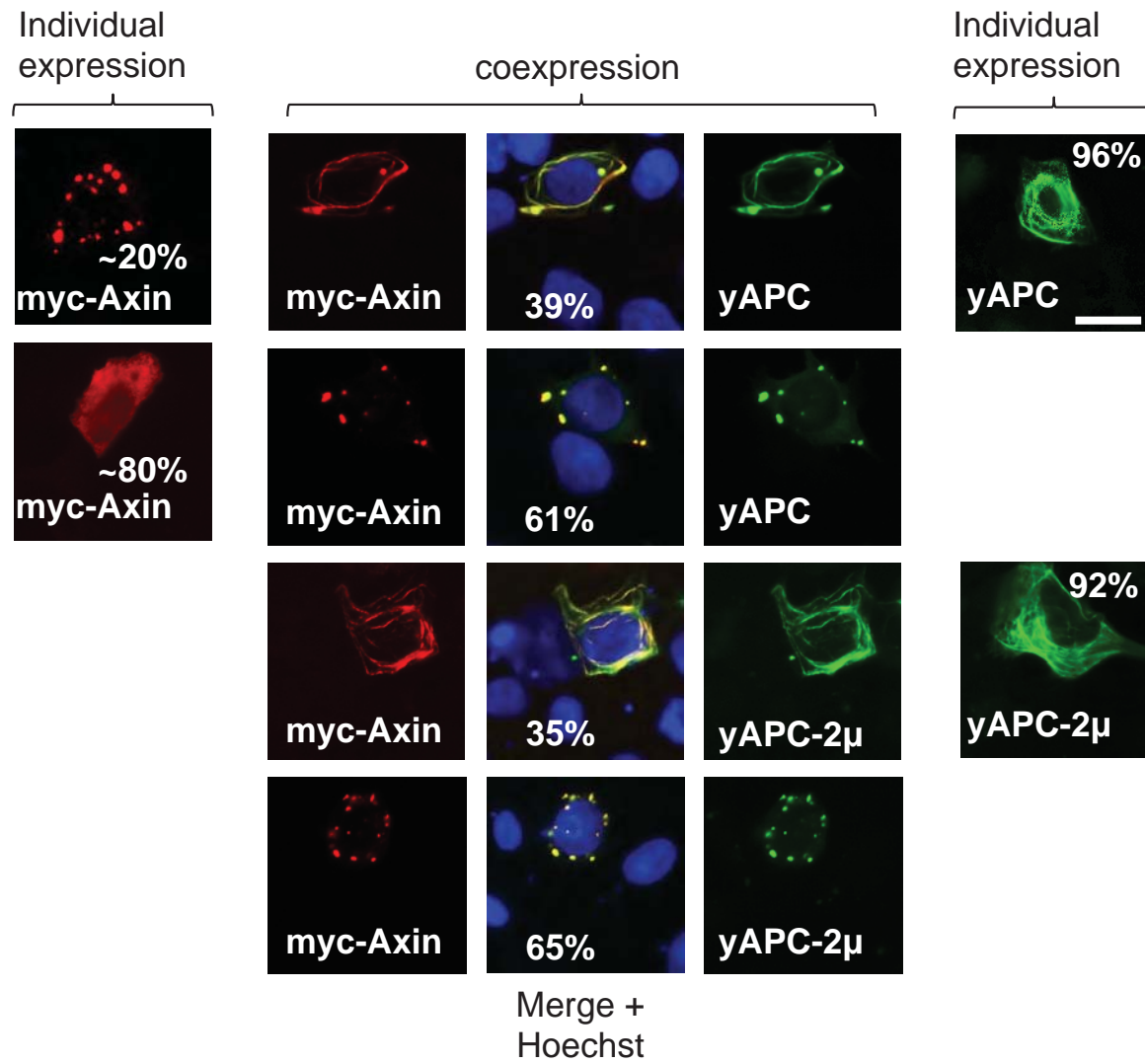

Supplement: Figure S10 — The 20R2 of full-length APC is not necessary to promote Axin co-oligomerisation. DLD1 cells were transiently transfected on day 1 with the indicated N-terminal YFP-tagged APC constructs or N-terminal myc-tagged Axin, either individually or in combination. The cells were fixed on day 3 and were stained with an anti-myc antibody and Hoechst dye. yAPC-2μ contains the mutations shown in figure 5A. Where applicable, the percentages indicate the proportion of different localisation patterns observed in the transfected cells. The imaging parameters were identical for each type of tag. Bar, 10 μM. (PDF) [file pone.0094413.s010.pdf]
