# Supplementary material for: Different Roles for the Axin Interactions with the SAMP versus the Second Twenty Amino Acid Repeat of Adenomatous Polyposis Coli
Source: PLoS One. 2014 Apr 10;9(4):e94413. doi: 10.1371/journal.pone.0094413 (PMC3983206; doi:10.1371/journal.pone.0094413)
Supplement: Figure S3 — Comparison of the 20R2 sequences in APC and APCL from different species. Residues common to all 20R2 sequences are highlighted in red. (PDF) [file pone.0094413.s003.pdf]

Figure S3.

APC Homo sapiens	KSPPEHYVQ**ETP**LMF**SR**CT**S**VS**SL**DSFESRSIASS
APC Taeniopygia guttata (zebra finch)	KSPPEHYVQ**ETP**LMF**SR**CT**S**VS**SL**DSFESRSIASS
APC Bos taurus (cattle)	KSPPEHYVQ**ETP**LMF**SR**CT**S**VS**SL**DSFESRSIASS
APC Xenopus laevis (African clawed frog)	KSPPEHYVQ**ETP**LMF**SR**CT**S**GS**SL**DSFESHSIASS
APC Drosophila melanogaster (fruit fly)	PPTQANSAL**ETP**LMF**SR**RS**S**MD**SL**VHDPDVDVANC
APC Danio rerio (zebrafish)	KSPPEHYVQ**ETP**LMF**SR**CT**S**VS**SL**DSFESHSIASS
APC Gallus gallus (chicken)	KSPPEHYVQ**ETP**LMF**SR**CT**S**VS**SL**DSFESRSIASS
APC Ornithorhynchus anatinus (platypus)	KSPPEHYVQ**ETP**LMF**SR**CT**S**VS**SL**DSFESRSIASS
APC Oryctolagus cuniculus (rabbit)	KSPPEHYVQ**ETP**LMF**SR**CT**S**VS**SL**DSFESRSIASS
APC Heterocephalus glaber (naked mole-rat)	KSPPEHYVQ**ETP**LMF**SR**CT**S**VS**SL**DSFESRSIASS
APC Oreochromis niloticus (Nile tilapia)	SPPEQPYAQ**ETP**LMF**SR**CT**S**VS**SL**ESFSTSSIASS
APC Ciona intestinalis (vase tunicate)	GTMTPKGYQ**ETP**MMF**SR**CS**S**MC**SL**SSFEAPSVQSQ
APCL Homo sapiens	SSSSENYVQ**ETP**LVL**SR**CS**S**VS**SL**GSFESPSIASS
APCL Drosophila melanogaster (fruit fly)	PPTQANSAL**ETP**LMF**SR**RS**S**MD**SL**VHDPDVDVANC
APCL Xenopus laevis (African clawed frog)	KSPPEHYVQ**ETP**LMF**SR**CT**S**GS**SL**DSFESHSIASS
APCL Heterocephalus glaber (naked mole-rat)	SSSSENCVQ**ETP**LVL**SR**CS**S**VS**SL**GSFESPSIASS
APCL Bos taurus (cattle)	SSSSENCVQ**ETP**LVL**SR**CS**S**VS**SL**GSFESPSIASS
APCL Oreochromis niloticus (Nile tilapia)	SSSSENYIH**ETP**LVM**SR**CS**S**VS**SL**GSFESPSIASS
APCL Danio rerio (zebrafish)	SSSSDNYIH**ETP**LVM**SR**CS**S**VS**SL**GSFESPSIASS
APCL Gallus gallus (chicken)	SSSSENYIQ**ETP**LVM**SR**CS**S**VS**SL**GSFESPSIASS
