# Supplementary material for: Different Roles for the Axin Interactions with the SAMP versus the Second Twenty Amino Acid Repeat of Adenomatous Polyposis Coli
Source: PLoS One. 2014 Apr 10;9(4):e94413. doi: 10.1371/journal.pone.0094413 (PMC3983206; doi:10.1371/journal.pone.0094413)
Supplement: Figure S4 — The 20R2 from a short internal APCL fragment is required to inhibit Axin oligomerisation. DLD1 cells were transiently transfected on day 1 with the N-terminal YFP-labelled APCL constructs or N-terminal flag-tagged Axin, either individually or in combination. The cells were fixed on day 3 and were stained with an anti-flag antibody and Hoechst dye. Where applicable, the percentages indicate the proportion of different localisation patterns observed in the transfected cells. The imaging parameters were identical for each type of tag. Bar, 10 μM. (PDF) [file pone.0094413.s004.pdf]

Figure S4.

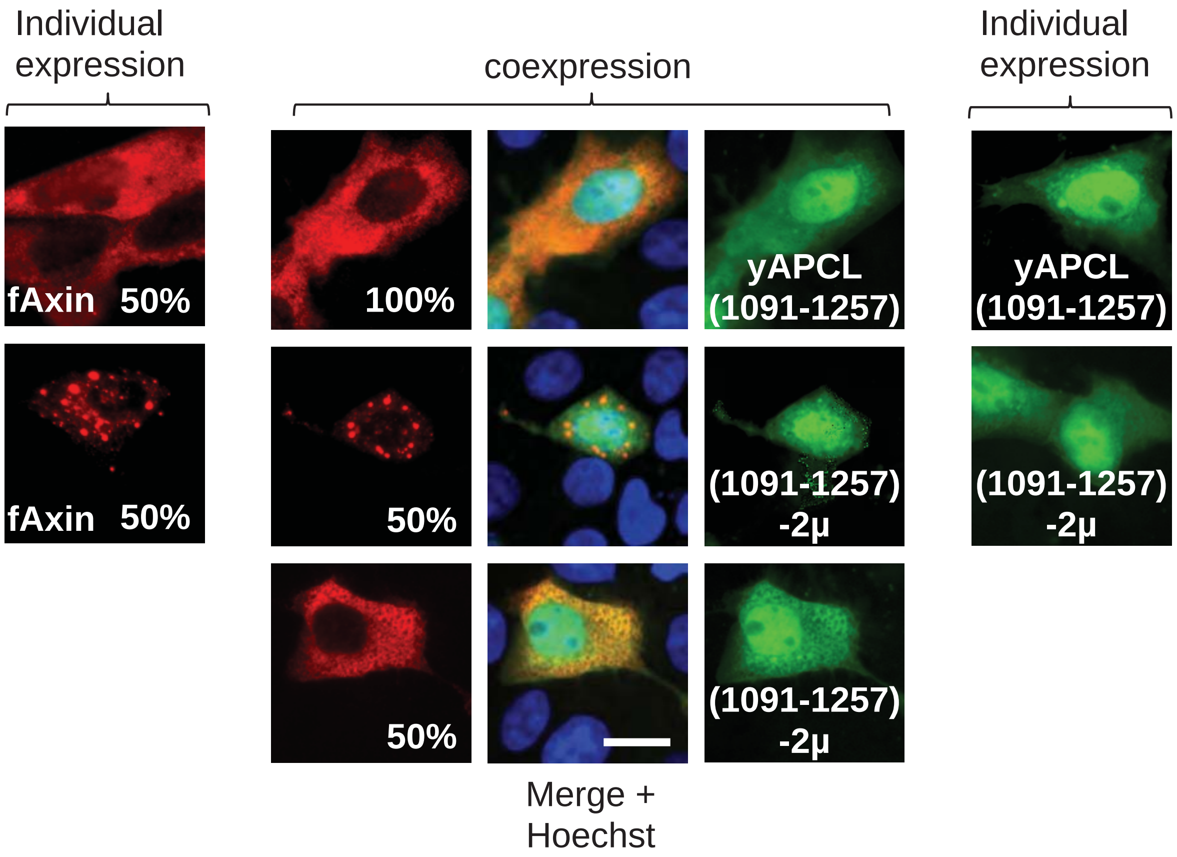
Individual expression
coexpression
Individual expression
fAxin 50%
100%
yAPCL (1091-1257)
yAPCL (1091-1257)
fAxin 50%
50%
(1091-1257) -2μ
(1091-1257) -2μ
50%
(1091-1257) -2μ
Merge + Hoechst
