# Supplementary material for: Different Roles for the Axin Interactions with the SAMP versus the Second Twenty Amino Acid Repeat of Adenomatous Polyposis Coli
Source: PLoS One. 2014 Apr 10;9(4):e94413. doi: 10.1371/journal.pone.0094413 (PMC3983206; doi:10.1371/journal.pone.0094413)
Supplement: Figure S11 — Pairwise comparison of the 20Rs of APC and APCL. Red indicates amino acids common to the 20R2s of APC and APCL from various species (fig. S3), red and blue represent the residues common to the 20R2s of human APC and APCL and the bold black letters indicate the amino acids common within each homologous 20R pair. The asterisks indicate residues from the 20R2 of APCL that are important for Axin binding and β-catenin degradation. Bar, 10 μM. (PDF) [file pone.0094413.s011.pdf]

Figure S11

```
                                    1155 1160 1164 1167
                                       *    *   *  *
APC2      VEFSSGAKSPSKSGAQTPKSPPEHYVQE-TPLMFSRCTSVSSLDSFESRSIASSVQSEPCSGM
APCL2     PAPRRNRGRGLGVEDATPSSSSENYVQE-TPLVLSRCSSVSSLGSFESPSIASSIPSEPCSGQ

APC1      RSGQPQKAATCKVSSINQETIQTYCV-EDTPICFSRCSSLSSLSSAEDEIGCNQTTQEADSAN
APCL1     HLSKVPEKLAAAPLSVASKALQKLAAQE-GPLSLSRCSSLSSLSSAGRPGPSEGGDLDDSDSS

APC3      KQAAVNAAVQRVQVLPDADTLLHFA-TESTPDGFSCSSSLSALSLDEPFIQKDVELRIMPPVQ
APCL3     RFLDIADCRERCRLPSELDAGSVRFTVEKPDENFSCASSLSALALHEHYVQQDVELRLLPSAC

APC4      PSQNRLQPQKHVSFTPGDDMPRVYCV-EGTPINFSTATSLSDLTIESPPNELAAGEGVRGGAQ
APCL4     ALPVPVYMLVPAPAPAQEDDSCTDSA-EGTPVNFSSAASLSDETLQGPPRDQPGGPAGRQRPT

APC5      FNDKLPNNEDRVRGSFAFDSPHHYTPIEGTPYCFSRNDSLSSLDFDDDDVDLSREKAELRKAK
APCL5     RKEAPAPSKAAPAAPPPARTQPSLIADE-TPPCYSLSSSASSLSEPEPSEPPAVHPRGREPAV

APC6      STFPQSSKDIPDRGAATDEKLQNFAI-ENTPVCFSHNSSLSSLSDIDQENNNKENEPIKETEP

APC7      ETEPPDSQGEPSKPQASGYAPKSFHV-EDTPVCFSRNSSLSSLSIDSEDDLLQECISSAMPKK
```
